# Supplementary material for: Cryo-EM structures of human zinc transporter ZnT7 reveal the mechanism of Zn2+ uptake into the Golgi apparatus
Source: Nat Commun. 2023 Aug 8;14:4770. doi: 10.1038/s41467-023-40521-5 (PMC10409766; doi:10.1038/s41467-023-40521-5)
Supplement: Supplementary file 1 — Supplementary Information [file 41467_2023_40521_MOESM1_ESM.pdf]

Supplementary Information

Cryo-EM structures of human zinc transporter ZnT7 reveal the mechanism of Zn^{2+} uptake into the Golgi apparatus

Bui Ba Han[1], Satoshi Watanabe[1], Norimichi Nomura, Kehong Liu, Tomoko Uemura, Michio Inoue, Akihisa Tsutsumi, Hiroyuki Fujita, Kengo Kinoshita, Yukinari Kato, So Iwata, Masahide Kikkawa & Kenji Inaba[2]

[1]Contributed equally to this work

[2]Corresponding author: kenji.inaba.a1@tohoku.ac.jp

The PDF file includes:

Supplementary Figures 1 to 23

Supplementary Tables 1 to 2

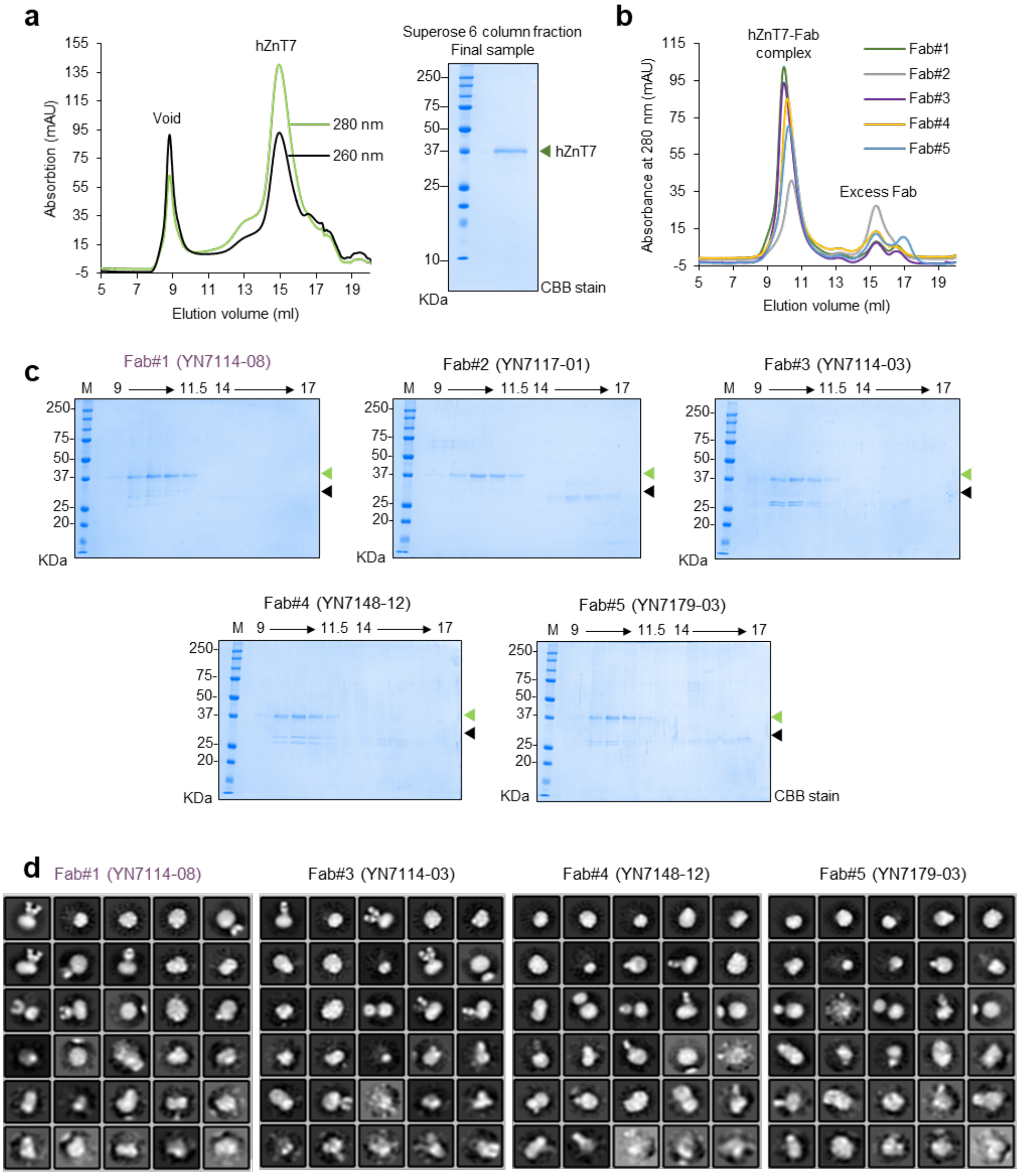

Supplementary Fig. 1 | Purifications of hZnT7 and its complex with Fabs for Cryo-EM analysis. a, Representative chromatogram from Superose 6 Increase gel filtration of hZnT7 (left) and a Coomassie-stained SDS gel of the final hZnT7 sample (right). Independent experiments were repeated with similar results at least three times. **b,** Representative chromatogram from Superdex 200 gel filtration of hZnT7 in complex with Fab fragments (#1 to #5) made from selected monoclonal antibodies. **c,** Coomassie-stained SDS gels of the hZnT7 (green triangle)-Fab (black triangle) complex separated by gel filtration chromatography shown in panel **b**. Independent experiments were repeated with similar results at least three times. **d,** Representative negative stained images of the hZnT7-Fab complexes after 2D classification averaging. The source data underlying Supplementary Fig. 1c are provided as a Source Data file.

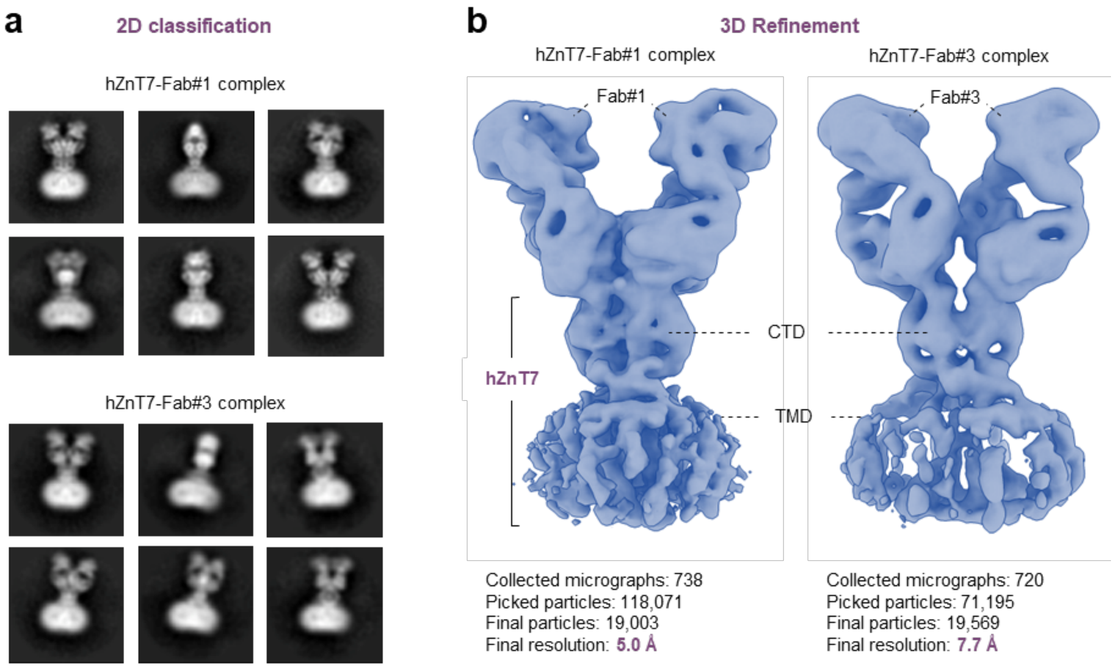

Supplementary Fig. 2 | Initial cryo-EM density maps of hZnT7-Fab#1 and -Fab#3 complexes measured by Talos Arctica TEM. a, Average 2D classification of the hZnT7-Fab#1 (top) and hZnT7-Fab#3 (bottom) complexes. **b,** Cryo-EM density maps of the hZnT7-Fab#1 complex (left) at 5.0 Å-resolution and of the hZnT7-Fab#3 complex (right) at 7.7 Å-resolution, both shown at contour levels of 0.025σ in UCSF ChimeraX. TMD, transmembrane domain; CTD, cytosolic domain.

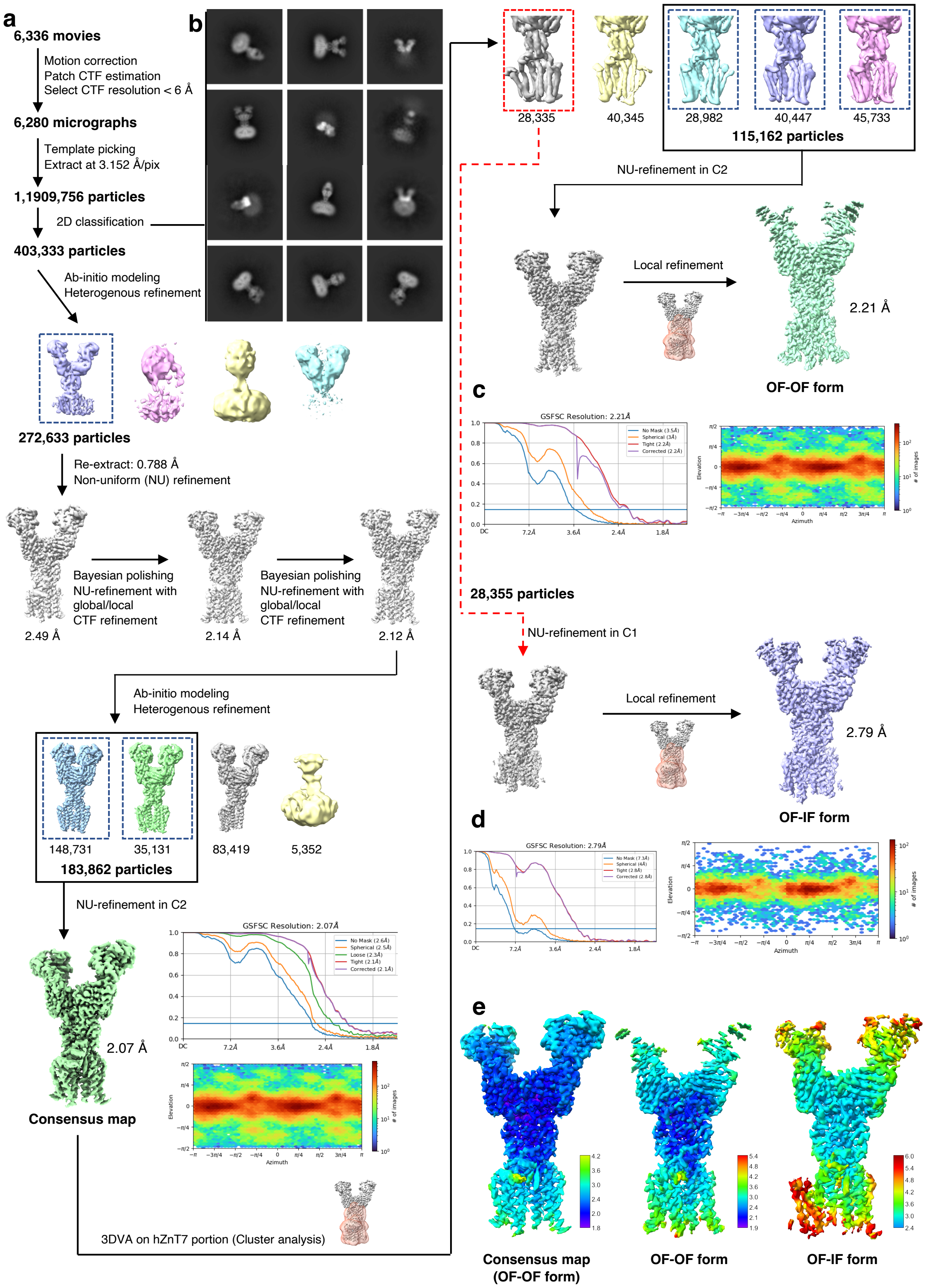

a
6,336 movies
Motion correction
Patch CTF estimation
Select CTF resolution < 6 Å
6,280 micrographs
Template picking
Extract at 3.152 Å/pix
1,1909,756 particles
2D classification
403,333 particles
Ab-initio modeling
Heterogenous refinement
272,633 particles
Re-extract: 0.788 Å
Non-uniform (NU) refinement
Bayesian polishing
NU-refinement with
global/local
CTF refinement
2.49 Å
2.14 Å
2.12 Å
Ab-initio modeling
Heterogenous refinement
148,731
35,131
83,419
5,352
183,862 particles
NU-refinement in C2
2.07 Å
Consensus map
GSFSC Resolution: 2.07Å
3DVA on hZnT7 portion (Cluster analysis)
b
28,335
40,345
28,982
40,447
45,733
115,162 particles
NU-refinement in C2
Local refinement
2.21 Å
OF-OF form
c
GSFSC Resolution: 2.21Å
28,355 particles
NU-refinement in C1
Local refinement
2.79 Å
OF-IF form
d
GSFSC Resolution: 2.79Å
e
Consensus map
(OF-OF form)
OF-OF form
OF-IF form

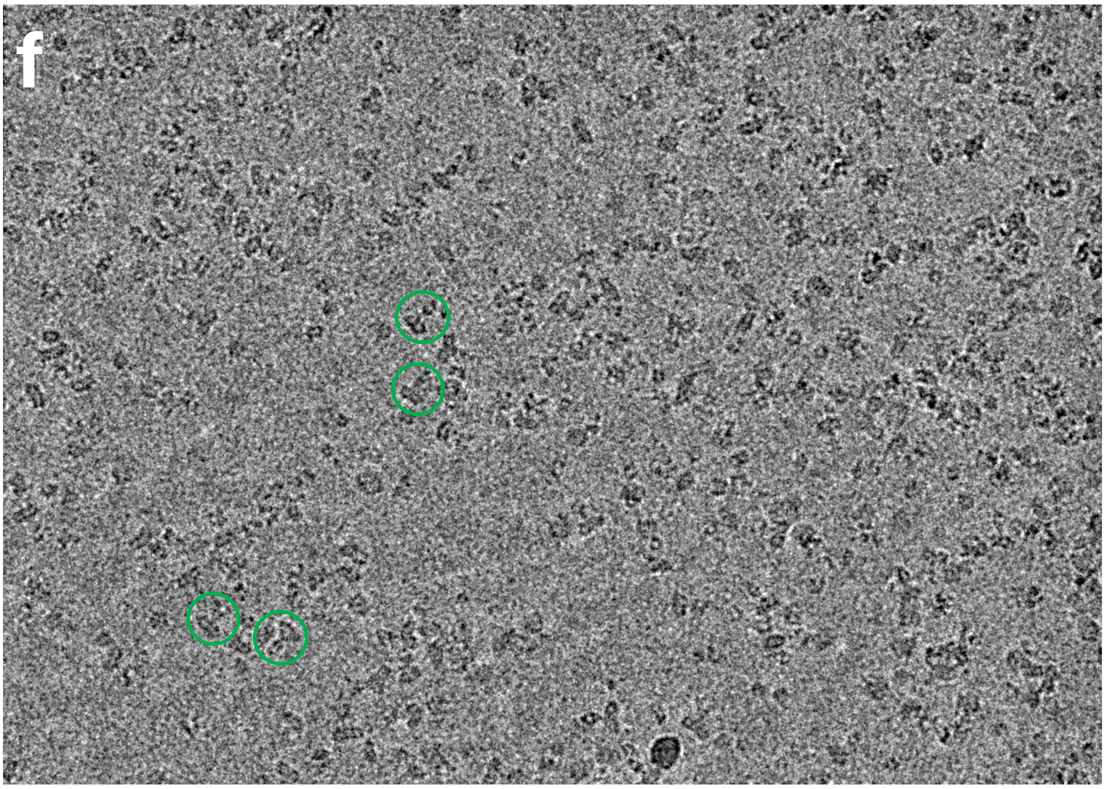

Supplementary Fig. 3 | Cryo-EM analysis of the hZnT7-Fab#1 complex prepared in the presence of 1 mM EDTA. a, Workflow of cryo-EM data processing and map refinement for the hZnT7-Fab#1 complex prepared in the presence of 1 mM EDTA. A representative cryo-EM micrograph after motion correction is shown in panel **f**. Details are provided in the Methods section. **b,** Representative 2D classification images of the hZnT7-Fab#1 complex analyzed by cryoSPARC. **c,** Gold-standard FSC plots of hZnT7-Fab#1 complex in OF-OF form from cryoSPARC (left). The blue line represents the gold-standard 0.143 FSC cutoff, which indicates a nominal resolution of 2.21 Å. Angular distribution calculated in cryoSPARC for hZnT7-Fab#1 complex particle projections (right). Heat map shows number of particles for each viewing angle (less = blue, more = red). **d,** Gold-standard FSC plots of hZnT7-Fab#1 complex in IF-OF form from cryoSPARC (left). The blue line represents the gold-standard 0.143 FSC cutoff, which indicates a nominal resolution of 2.79 Å. Angular distribution calculated in cryoSPARC for hZnT7-Fab#1 complex particle projections (right). Heat map shows number of particles for each viewing angle (less = blue, more = red). **e,** Local-resolution map of the hZnT7-Fab#1 complex in the OF-OF form (Consensus map, left), OF-OF form (middle) and IF-OF form (right) calculated by cryoSPARC. The bar on the right side of each map indicates local resolution in Å. **f,** A representative cryo-EM micrograph of the hZnT7-Fab#1 complex prepared in the presence of 1 mM EDTA. The hZnT7-Fab#1 complex particles viewed from the side are indicated by green circles.

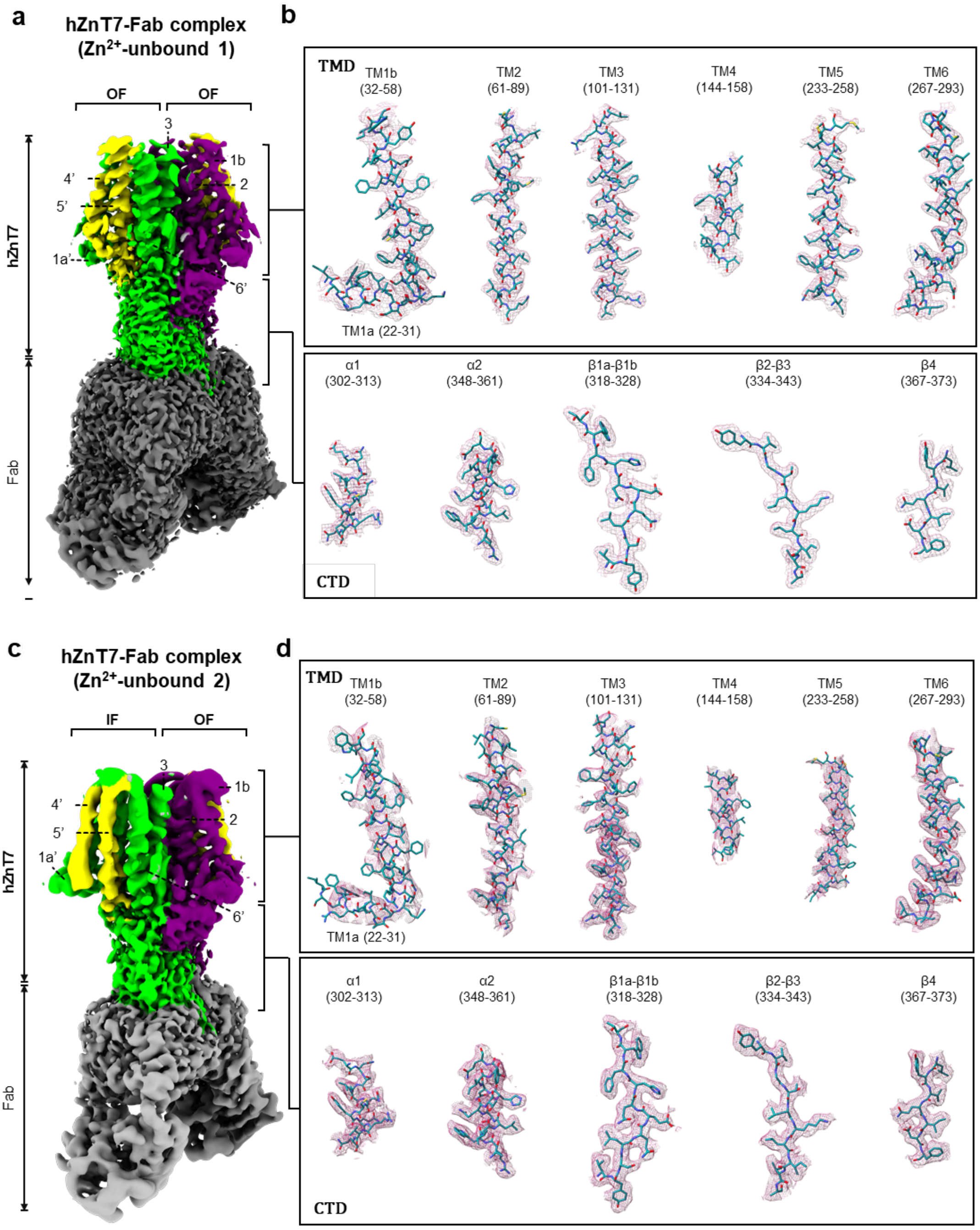

Supplementary Fig. 4 | Cryo-EM density of the hZnT7-Fab#1 complex prepared in the presence of 1 mM EDTA. a, Side view of the cryo-EM map of Zn^{2+}-unbound OF-OF homodimer of hZnT7 in complex with Fab. Chain A (violet), chain B (green), TMs 4-5 (yellow) and Fab (gray) are highlighted. Numbers indicate the transmembrane (TM) helix number from the N-terminus. **b,** Cryo-EM density maps of TM1-6 and the cytosolic domain (CTD) of the Zn^{2+}-unbound hZnT7 homodimer (OF-OF form). **c,** Side view of the cryo-EM map of Zn^{2+}-unbound IF-OF heterodimer of hZnT7 in complex with Fab. Chain A (violet), chain B (green), TMs 4-5 (yellow) and Fab (gray) are highlighted. Numbers indicate the transmembrane (TM) helix number from the N-terminus. **d,** Cryo-EM density maps of TM1-6 and the cytosolic domain (CTD) of the IF protomer in the Zn^{2+}-unbound hZnT7 heterodimer (IF-OF form).

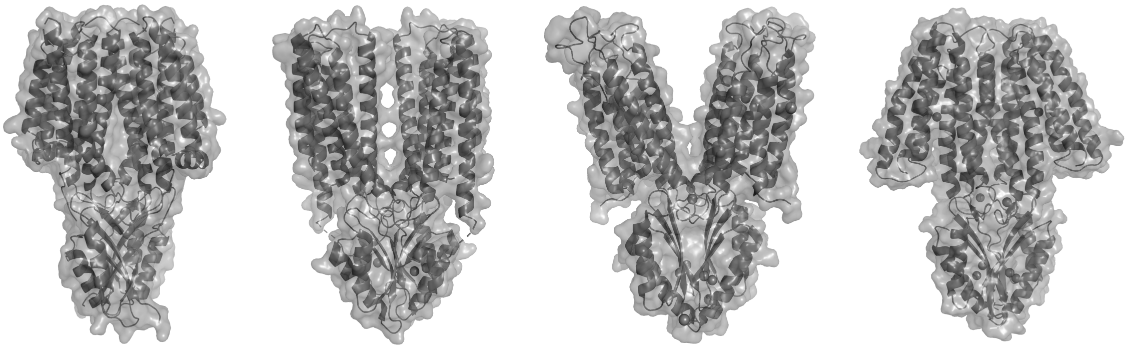

Supplementary Fig. 5 | Comparison of cryo-EM maps and overall structures between hZnT7, hZnT8 and bacterial YiiP (EcYiiP and SoYiiP). Comparison of the overall structures of hZnT7 (this study) and hZnT8 (PDB ID: 6XPD) in Zn^{2+}-unbound OF-OF form, EcYiiP in Zn^{2+}-bound OF-OF form (PDB ID: 3H90) and SoYiiP (PDB ID: 7KZZ) in Zn^{2+}-bound IF-IF form. Backbones and surfaces are represented by dark gray cartoon and light gray surface, respectively.

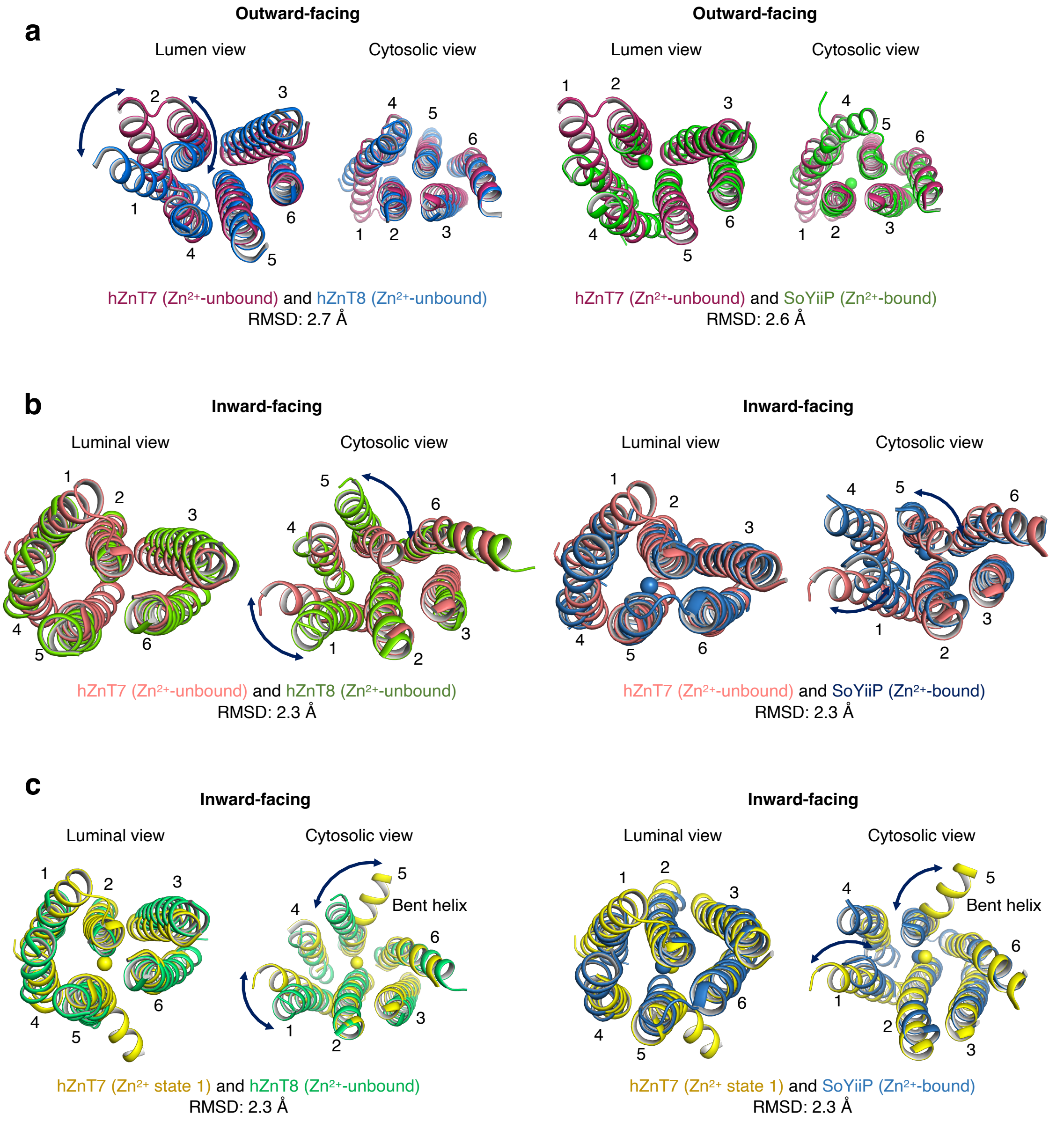

Supplementary Fig. 6 | Structural comparisons of the TMDs between hZnT7, hZnT8 and bacterial YiiP. a, Structural alignment of the TMD of Zn^{2+}-unbound hZnT7 (red), hZnT8 (blue, Zn^{2+}-unbound, PDB ID: 6XPD) and EcYiiP (green, Zn^{2+}-bound, PDB ID: 3H90) protomers in the OF conformation. The black double-headed arrows indicate large difference in the orientations of TM1b and TM2 between hZnT7 and hZnT8. Numbers indicate the TM helix number from the N-terminus. **b,** Structural alignment of the TMD of Zn^{2+}-unbound hZnT7 (salmon), hZnT8 (green, Zn^{2+}-unbound, PDB ID: 6XPF) and SoYiiP (blue, Zn^{2+}-bound, PDB ID: 7KZZ) protomers in the IF conformation. The black double-headed arcs indicate large difference in the orientations of TM1b and TM5 between hZnT7, hZnT8 and SoYiiP. **c,** Structural alignment of the TMD of the IF protomers in hZnT7 in Zn^{2+} state 1 (yellow), Zn^{2+}-unbound hZnT8 (green, PDB ID: 6XPF) and Zn^{2+}-bound SoYiiP (blue, PDB ID: 7KZZ). The black double-headed arrows indicate large difference in the orientations of TM1 and TM5 between hZnT7 and hZnT8, and the bending of TM5 seen in Zn^{2+} state 1 of hZnT7.

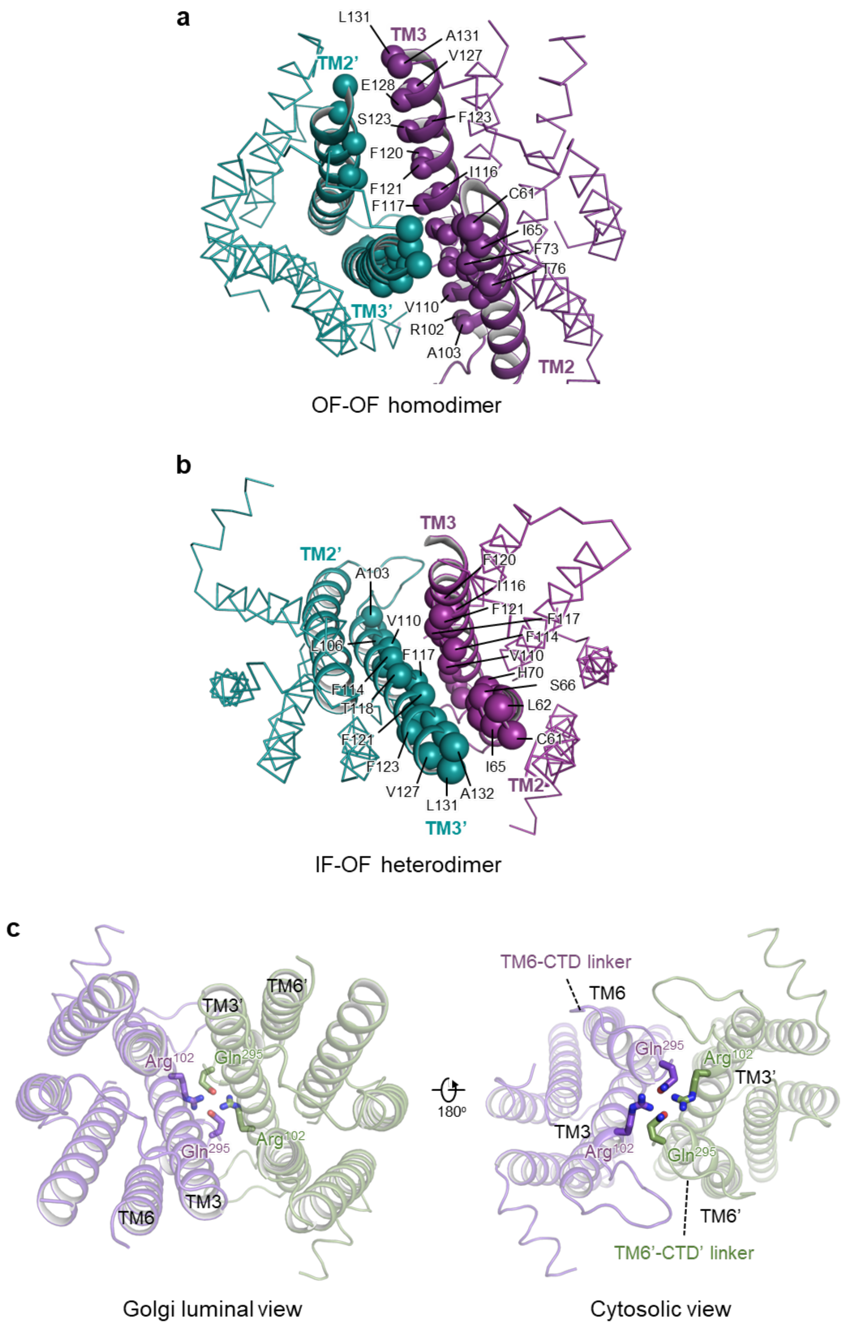

Supplementary Fig. 7 | Interactions between the TMDs in the Zn^{2+}-unbound hZnT7 homodimer and heterodimer. a, TMD of Zn^{2+}-unbound hZnT7 homodimer (upper) composed of two OF protomers (OF-OF form). TMs 2-3 and TMs 2'-3' are represented by cartoon, and the other helices are shown as ribbons. Residues involved in the dimer interface are shown as spheres. The CTD is not shown for clarity. **b,** TMD of Zn^{2+}-unbound hZnT7 heterodimer (upper) composed of IF and OF protomers (IF-OF form). TM helices and residues involved in the dimer interface are shown as in panel **a**. The CTD is not shown for clarity. **c,** Golgi luminal (left) and cytosolic (right) views of the interlock formed between Arg102 and Gln295 at the dimer interface. The residues involved in this interlock are represented by sticks. The CTD is not shown for clarity.

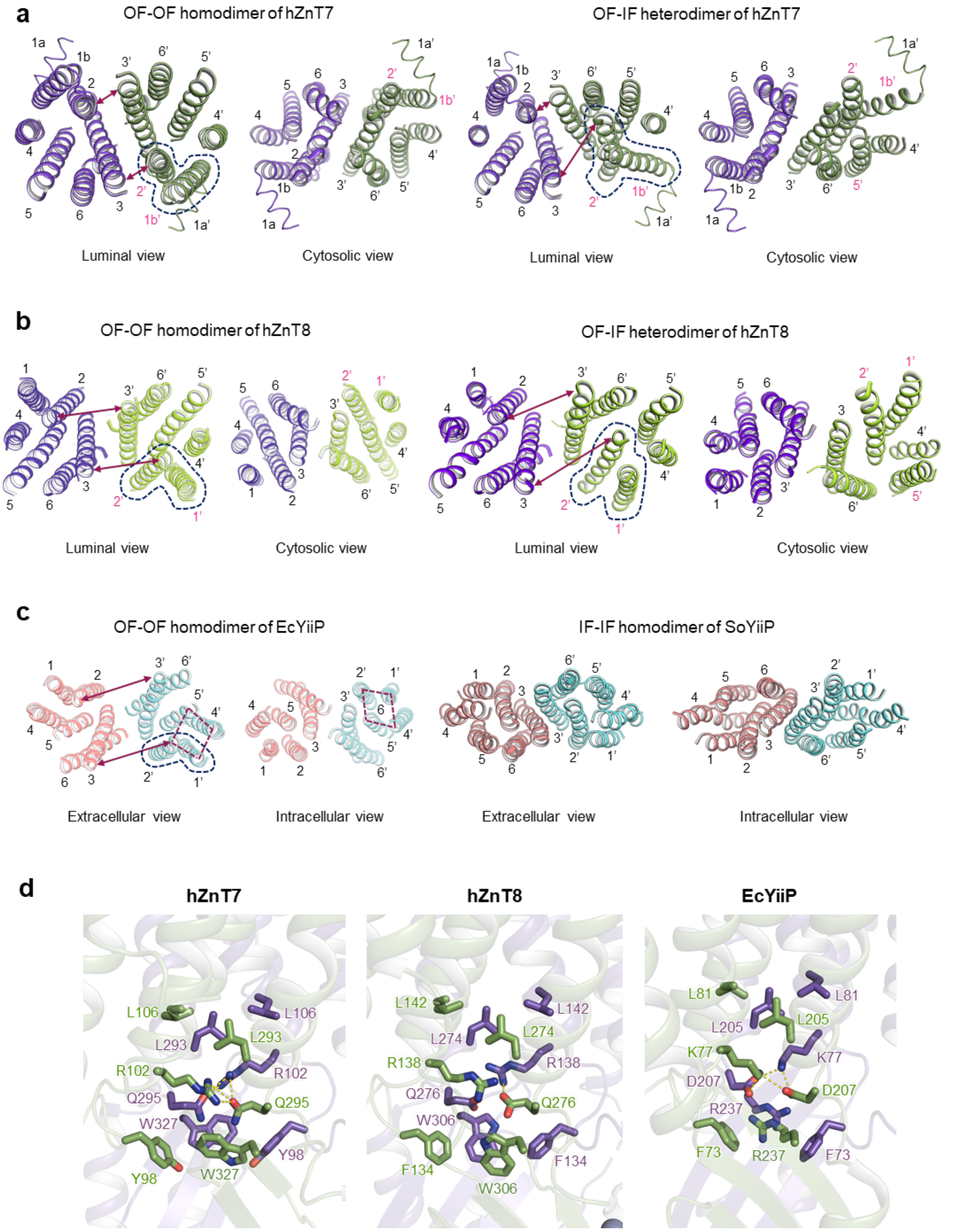

Supplementary Fig. 8 | Structural comparisons between hZnT7, hZnT8 and bacterial YiiP. a, Golgi luminal and cytosolic views of the TMD of Zn^{2+}-unbound hZnT7 homodimer composed of the OF protomers (OF-OF form, left) and of the Zn^{2+}-unbound hZnT7 heterodimer composed of the IF-OF protomers (IF-OF form, right). The CTD and TM loops are omitted for clarity. Numbers indicate the TM helix number from the N-terminus. The red double-headed arrows indicate the proximity between TM2 and TM3' or TM2' and TM3 at the dimer interface. The

black dotted enclosure indicates different orientations of TM1b’ and TM2’ between the OF and IF protomers. **b,** Golgi luminal and cytosolic views of the TMD in the OF-OF homodimer (left, PDB ID: 6XPD) and OF-IF heterodimer (right, PDB ID: 6XPF) of hZnT8. The CTD and TM loops are omitted for clarity. The red double-headed arrows indicate the large separation between TM2 (TM2’) and TM3’ (TM3). **c,** Extracellular and intracellular views of the TMD in the OF-OF homodimer of EcYiiP (left, PDB ID: 3H90) and the IF-IF homodimer of SoYiiP (right, PDB ID: 7KZZ). The CTD and TM loops are omitted for clarity. The red double-headed arrows indicate the large separation between TM2 (TM2’) and TM3’ (TM3) of different protomers. The black dotted enclosure indicates different orientations of TM1b’ and TM2’ between the OF and IF protomers. The red dotted rectangles indicate the TM helix bundle composed of TM1, TM2, TM4 and TM5 in the EcYiiP homodimer. **d,** Side view of the interlock formed at the dimer interface of hZnT7, hZnT8 and EcYiiP. The residues involved in this interlock are represented by sticks.

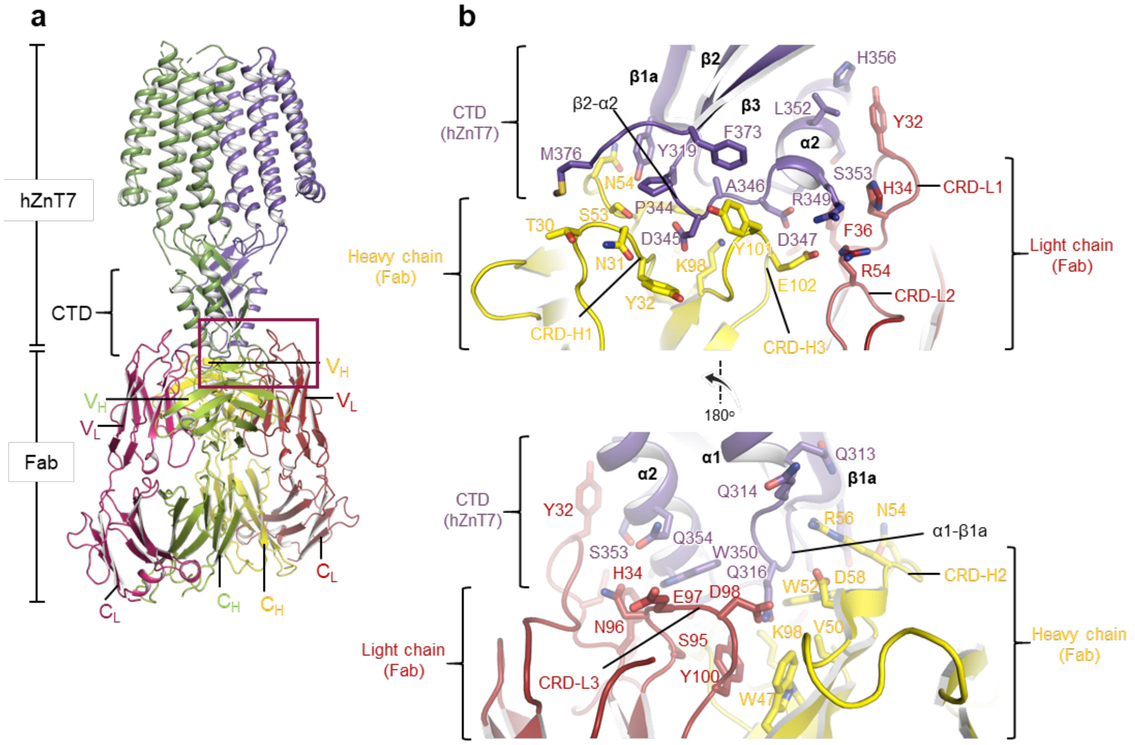

Supplementary Fig. 9 | Mode of interactions between the cytosolic domain (CTD) of hZnT7 and Fab#1. a, Cartoon representation of the Zn^{2+}-unbound hZnT7 homodimer complexed with Fab. The interface between the CTD and Fab is marked by a red square and highlighted in panel **b**. **b,** Highlighted view of the CTD-Fab interface. Residues involved in the CTD-Fab interactions are represented by sticks.

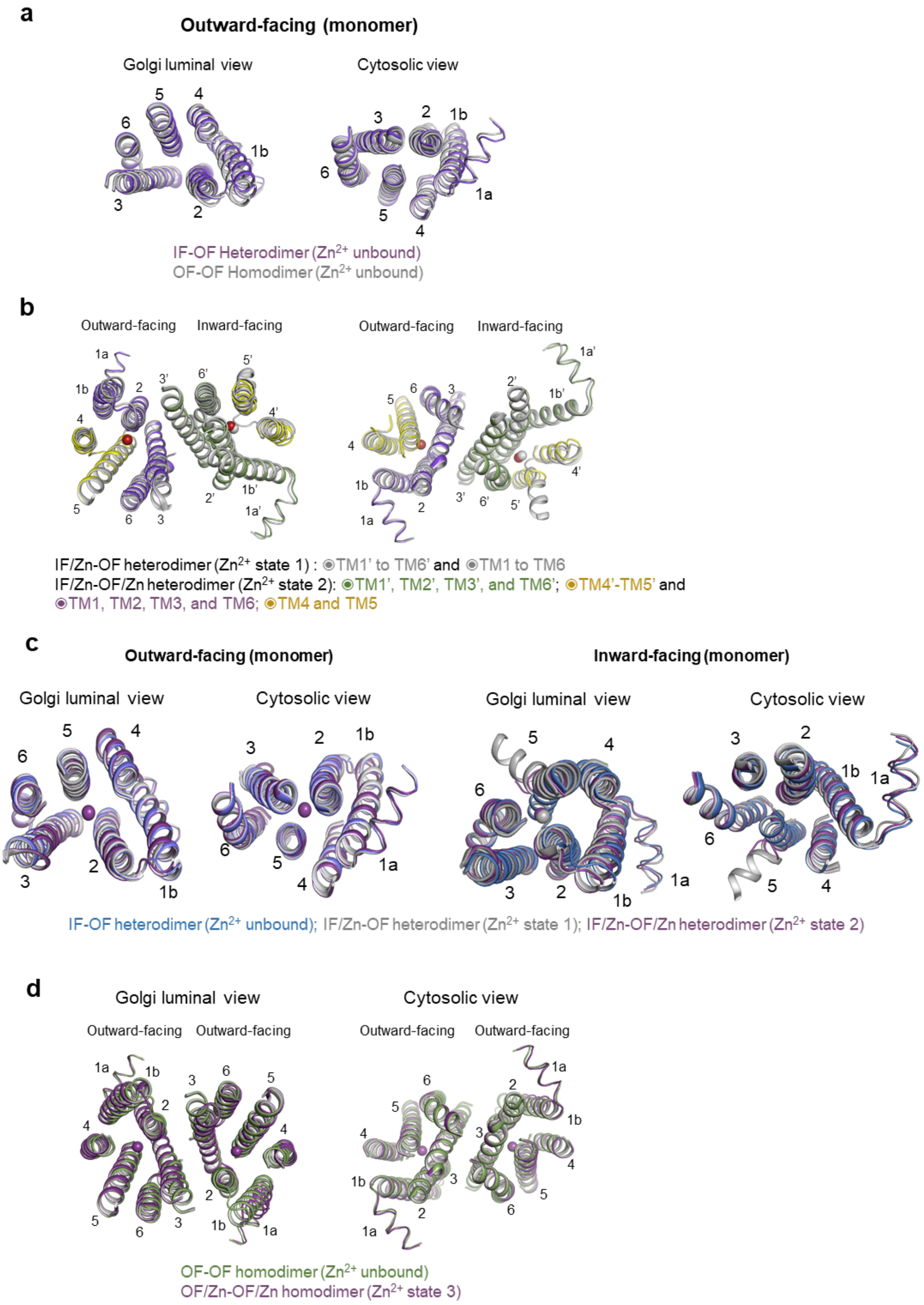

Supplementary Fig. 10 | Structural comparisons of the hZnT7 dimers. a, Structural alignment of the TMD in the Zn^{2+}-unbound OF protomer of the hZnT7 heterodimer (IF-OF form, violet) and homodimer (OF-OF form, gray) viewed from the Golgi luminal (left) and cytosolic (right) sides. For clarity, the CTD and TM loops are omitted. **b,** Structural alignment of the TMD in the hZnT7

heterodimers in Zn^{2+} state 1 (gray) and Zn^{2+} state 2 (color). Red spheres indicates bound Zn^{2+}. For clarity, the CTD and TM loops are omitted. **c,** Structural alignment of the TMD in the OF (left) and IF (right) protomers of hZnT7 in IF-OF form (blue), Zn^{2+} state 1 (gray) and Zn^{2+} state 2 (violet). Violet or gray spheres indicates bound Zn^{2+}. For clarity, the CTD and TM loops are not shown. **d,** Structural alignment of the hZnT7 TMD in the OF-OF form (green) and Zn^{2+} state 3 (violet) viewed from the Golgi luminal (left) and cytosolic (right) sides. For clarity, the CTD and TM loops are omitted.

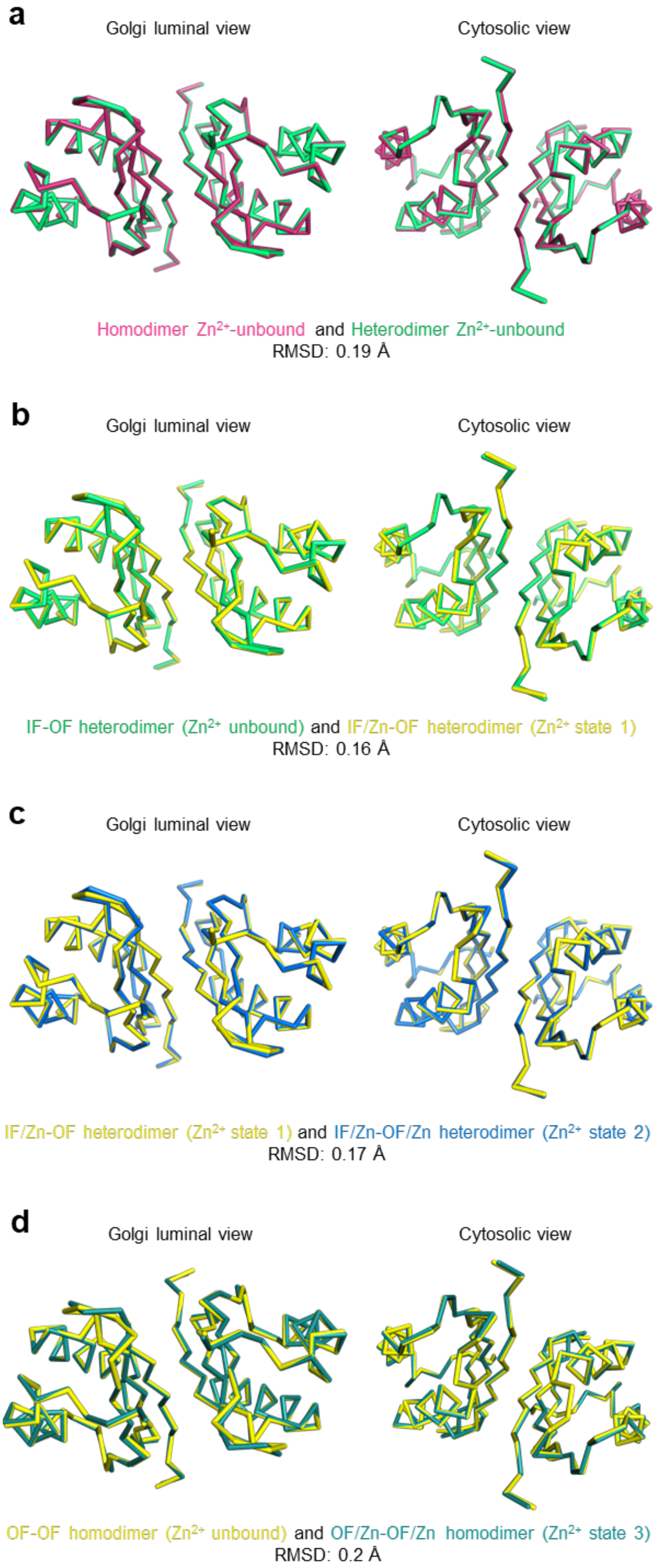
a
Golgi luminal view
Cytosolic view
Homodimer Zn2+-unbound and Heterodimer Zn2+-unbound
RMSD: 0.19 Å
b
Golgi luminal view
Cytosolic view
IF-OF heterodimer (Zn2+ unbound) and IF/Zn-OF heterodimer (Zn2+ state 1)
RMSD: 0.16 Å
c
Golgi luminal view
Cytosolic view
IF/Zn-OF heterodimer (Zn2+ state 1) and IF/Zn-OF/Zn heterodimer (Zn2+ state 2)
RMSD: 0.17 Å
d
Golgi luminal view
Cytosolic view
OF-OF homodimer (Zn2+ unbound) and OF/Zn-OF/Zn homodimer (Zn2+ state 3)
RMSD: 0.2 Å

Supplementary Fig. 11 | Structural comparisons of the CTDs of hZnT7 in different states. **a**, Structural alignment of the CTD between the Zn^{2+}-unbound hZnT7 homodimer (OF-OF form) and heterodimer (IF-OF form). **b**, Structural alignment of the CTD between the hZnT7 heterodimers in IF-OF form and Zn^{2+} state 1. **c**, Structural alignment of the CTD between the hZnT7 heterodimers in Zn^{2+} state 1 and Zn^{2+} state 2. **d**, Structural alignment of the CTD between the hZnT7 homodimers in OF-OF form and Zn^{2+} state 3. The TMD is not shown for clarity.

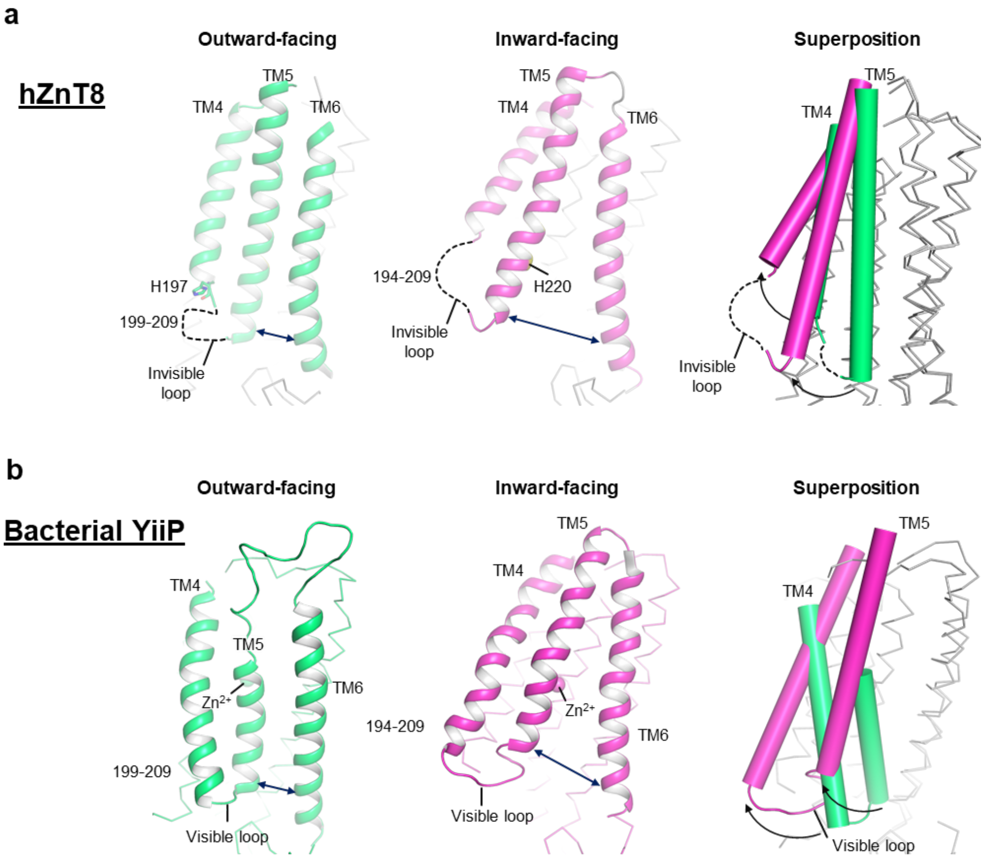

Supplementary Fig. 12 | Structural comparisons of TM4 and TM5 in the OF and IF conformations of hZnT8 and bacterial YiiP. **a**, Cartoon representation of TM4, TM5 and TM6 in the OF (green, left) and IF (magenta, middle) protomers of hZnT8 and their superimposition (right). For clarity, only TM4 and TM5 are shown as cylinders. The black arrows indicate the rotation of TM4 and TM5 during the conversion from the OF to IF conformations. **b**, Cartoon representation of TM4, TM5 and TM6 in the OF (green, left) and IF (magenta, middle) protomers of bacterial YiiP (left) and their superposition (right). For clarity, only TM4 and TM5 are shown as cylinders. The black arrows indicate the rotation of TM4 and TM5 during the conversion from the OF to IF conformations.

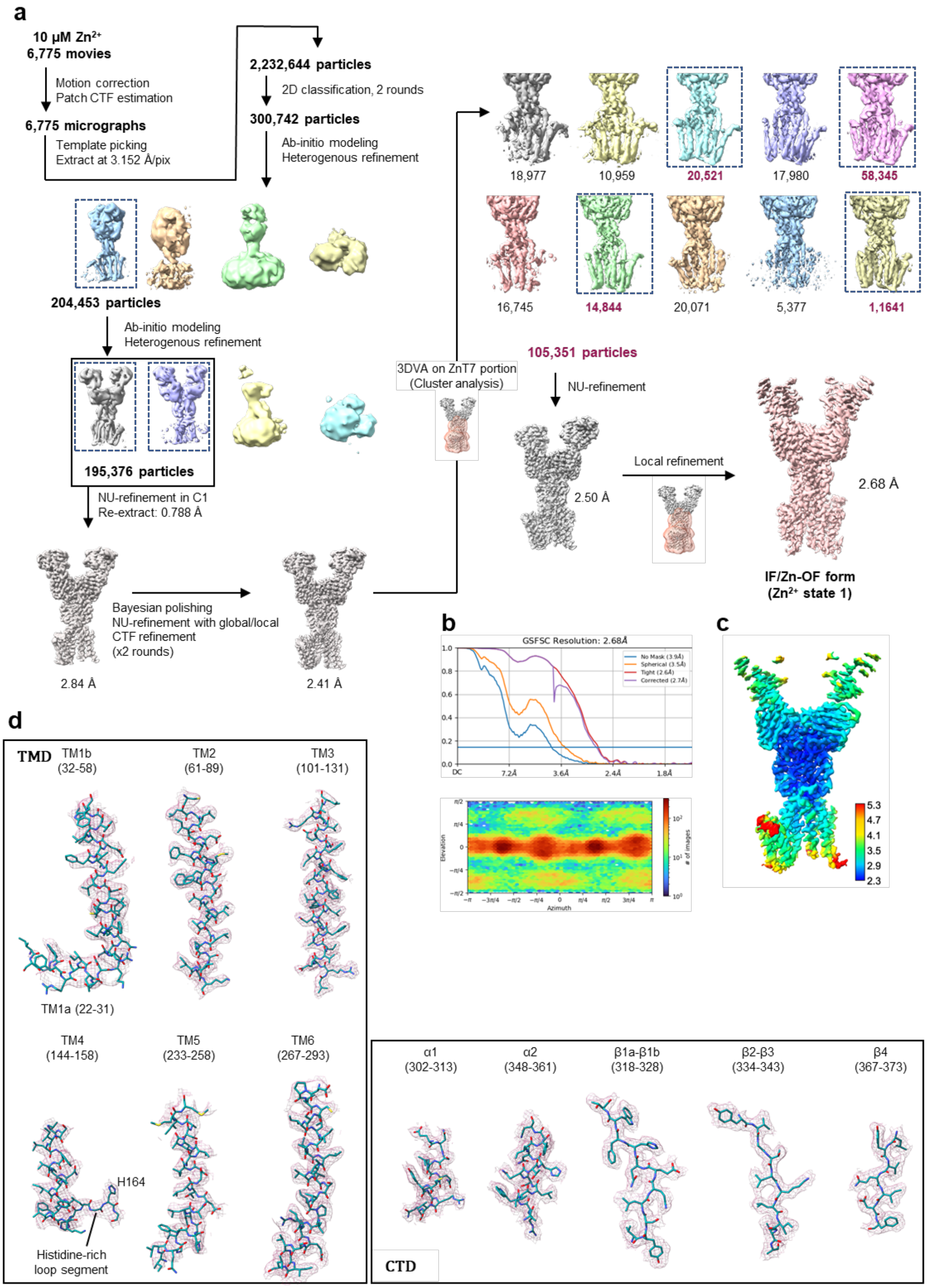

Supplementary Fig. 13 | Cryo-EM analysis of the hZnT7-Fab#1 complex in the presence of 10 μM Zn^{2+}. a, Workflow of cryo-EM data processing and map refinement for the hZnT7-Fab#1 complex in the presence of 10 μM Zn^{2+}. Details are provided in the Methods section. **b,** Gold-standard FSC plots of the hZnT7-Fab#1 complex in IF/Zn1-OF form (Zn^{2+}-state 1) from

cryoSPARC (up). The blue line represents the gold-standard 0.143 FSC cutoff, which indicates a nominal resolution of 2.68 Å. Angular distribution calculated in cryoSPARC for hZnT7-Fab#1 complex particle projections (bottom). Heat map shows number of particles for each viewing angle (less = blue, more = red). **c**, Local-resolution map of the hZnT7-Fab#1 complex in IF/Zn1-OF form (Zn^{2+} state 1) calculated by cryoSPARC. Bars on the right side indicate local resolution in Å. **d**, Cryo-EM density for TM 1-6 and the cytosolic domain (CTD) of the IF protomer in the IF/Zn1-OF heterodimer of hZnT7.

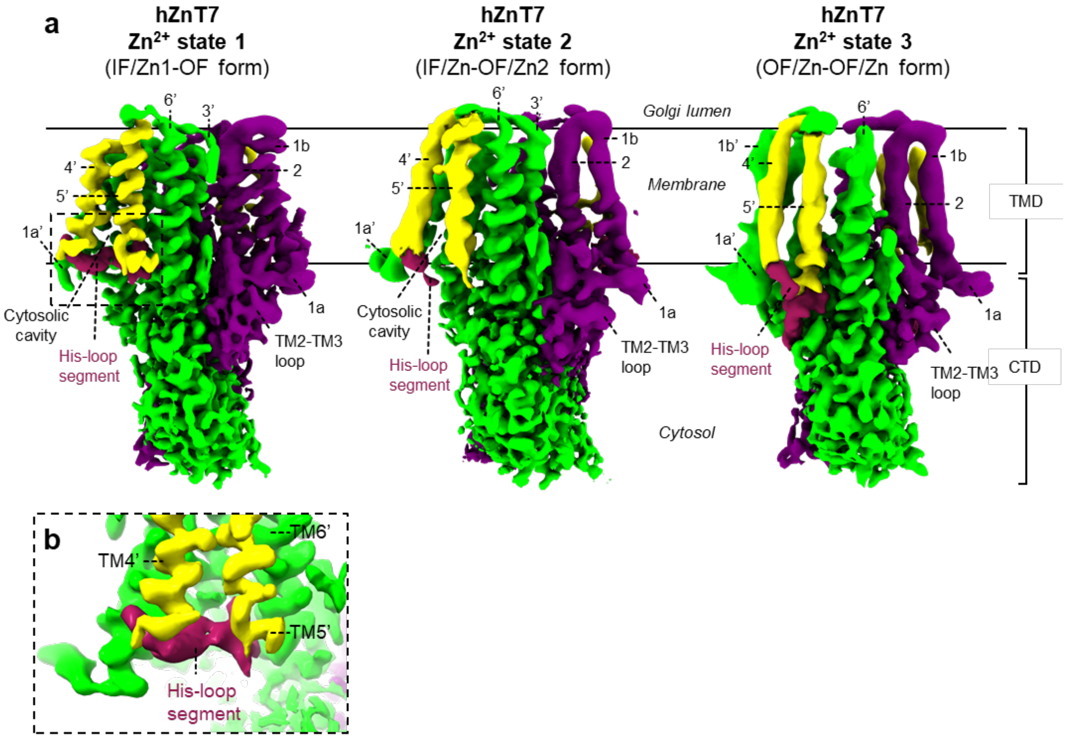

Supplementary Fig. 14 | Cryo-EM density maps of hZnT7 in Zn^{2+} states 1-3. a, Side view of the cryo-EM maps of hZnT7 Zn^{2+} state 1 (IF/Zn-OF form, left), Zn^{2+} state 2 (IF/Zn-OF/Zn form, middle) and Zn^{2+} state 3 (OF/Zn-OF/Zn form, right). Chain A (violet), chain B (green), TMs 4-5 (yellow) and the His-loop segment (magenta) are highlighted. Numbers indicate the transmembrane (TM) helix number from the N-terminus. TMD, transmembrane domain; CTD, cytosolic domain. The black dashed-line square indicates the region that is highlighted in panel (**b**). **b,** Zoomed-in view of the density of the visible His-loop segment (magenta) incorporated in the cytosolic cavity. Cryo-EM density of TM4 and TM5 is colored yellow, while that of TM1a, TM1b, TM2, TM3 and TM6 is colored light green.

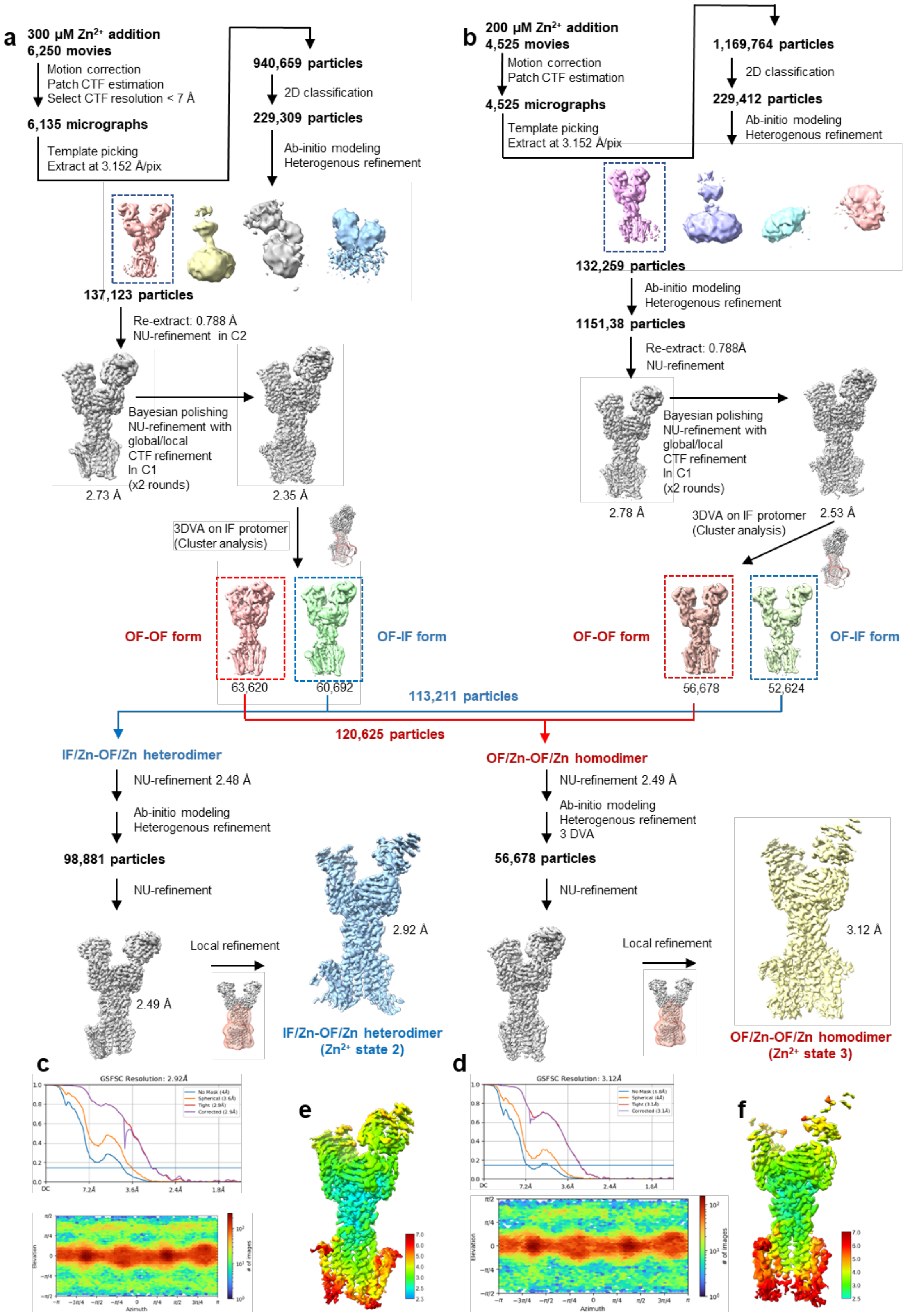

Supplementary Fig. 15 | Cryo-EM analysis of the hZnT7-Fab#1 complex with exogenously added 200 and 300 μM of Zn^{2+}. a, Workflow of cryo-EM data processing and map refinement for the hZnT7-Fab#1 complex in the presence of 200 μM Zn^{2+}. Details are provided in the

Methods section. **b,** Workflow of cryo-EM data processing and map refinement for the hZnT7-Fab#1 complex in the presence of 300 μM Zn^{2+}. Details are provided in the Methods section. **c,** Gold-standard FSC plots of hZnT7-Fab#1 complex in IF/Zn-OF form (Zn^{2+} state 2) from cryoSPARC (up). The blue line represents the gold-standard 0.143 FSC cutoff, which indicates a nominal resolution of 2.92 Å. Angular distribution calculated in cryoSPARC for hZnT7-Fab#1 complex particle projections (bottom). Heat map shows number of particles for each viewing angle (less = blue, more = red). **d,** Gold-standard FSC plots of hZnT7-Fab#1 complex in OF/Zn-OF/Zn form (Zn^{2+} state 3) from cryoSPARC (up). The blue line represents the gold-standard 0.143 FSC cutoff, which indicates a nominal resolution of 3.12 Å. Angular distribution calculated in cryoSPARC for hZnT7-Fab#1 complex particle projections (bottom). Heat map shows number of particles for each viewing angle (less = blue, more = red). **e,** Local-resolution map of the hZnT7-Fab#1 complex in the IF/Zn-OF form (Zn^{2+} state 2) calculated by cryoSPARC. The bar on the right side indicates local resolution in Å. **f,** Local-resolution map of the hZnT7-Fab#1 complex in the OF/Zn-OF/Zn form (Zn^{2+} state 3) calculated by cryoSPARC. The bar on the right side indicates local resolution in Å.

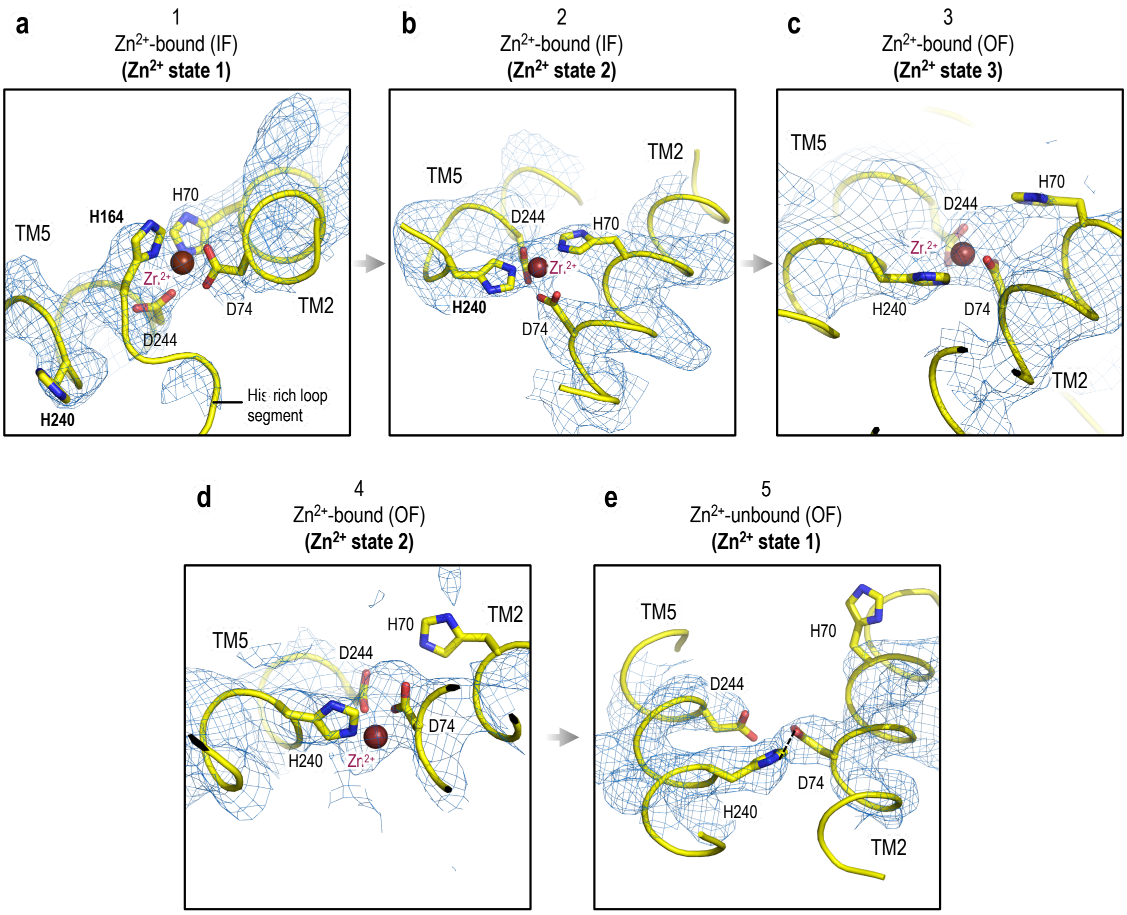

Supplementary Fig. 16 | Zn^{2+}-coordination structure of hZnT7. Another angle view of the Zn^{2+}-binding site in the IF protomer in Zn2^{+} state 1 (**a**) and Zn^{2+} state 2 (**b**), in the OF protomer in Zn^{2+} state 3 (**c**), Zn^{2+} state 2 (**d**) and Zn^{2+} state 1 (**e**). The red spheres indicate bound Zn^{2+}. The density map and Zn^{2+}-binding residues are represented by blue mesh and yellow sticks, respectively. The main chains are represented by yellow ribbons. Black dashed lines indicate the hydrogen bond between His240 and Asp74 (**e**). See also Fig. 6.

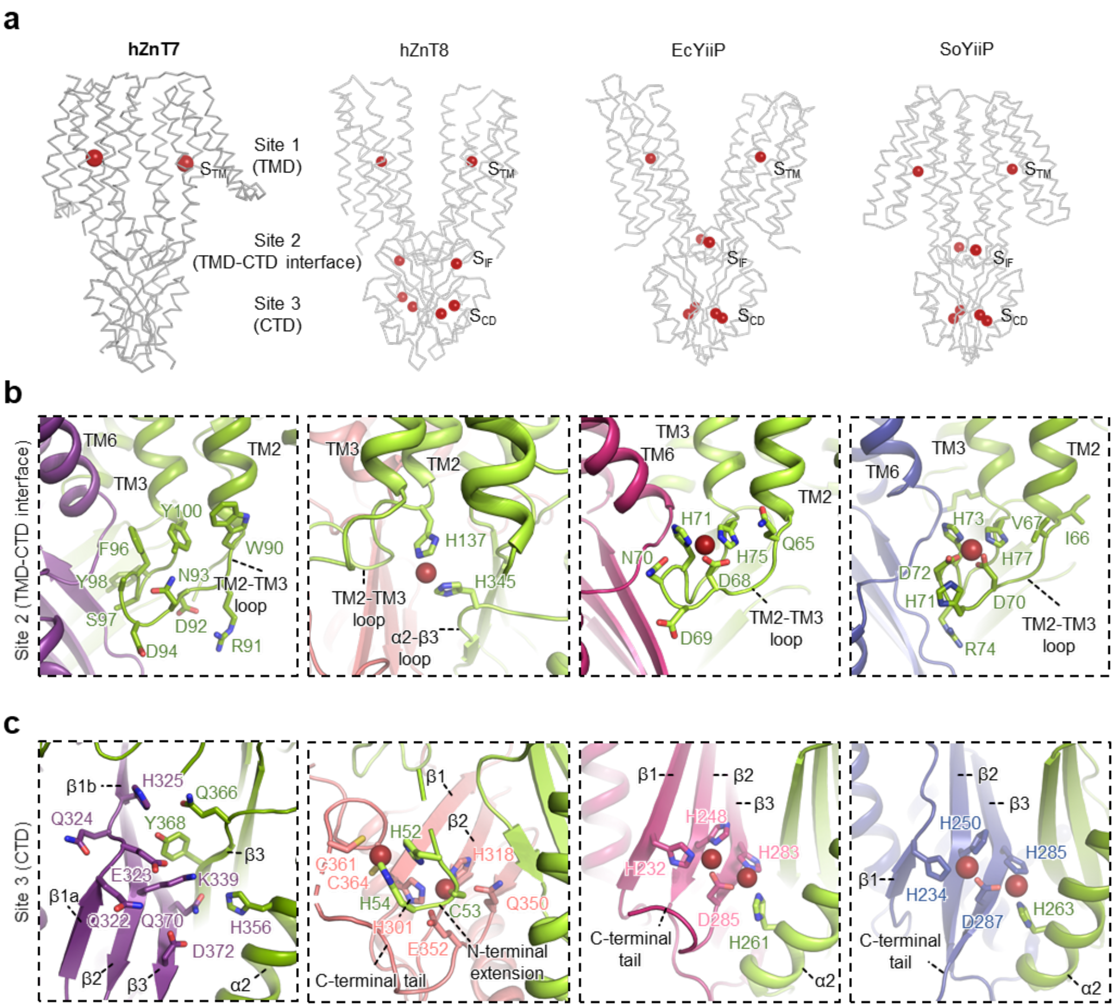

Supplementary Fig. 17 | Comparison of the Zn^{2+}-binding sites between hZnT7, hZnT8 and bacterial YiiP. a, Ribbon representations of hZnT7, hZnT8 and bacterial YiiP (EcYiiP and SoYiiP) with bound Zn^{2+} (red spheres). TMD, transmembrane domain; CTD, cytosolic domain. **b,** Close-up views of Zn^{2+}-binding sites around the TMD-CTD interface (Site 2) in hZnT7, hZnT8 and bacterial YiiP (EcYiiP and SoYiiP). Bound Zn^{2+} are shown as red spheres. Note that no Zn^{2+} is bound at Site 2 in hZnT7. **c,** Close-up views of Zn^{2+}-binding sites around the CTD (Site 3) in hZnT7, hZnT8 and bacterial YiiP (EcYiiP and SoYiiP). Bound Zn^{2+} are shown as red spheres. Note that no Zn^{2+} is bound at Site 3 in hZnT7.

```
                                                   ▼▼ ▼          ▼ ▼ ▼
hZnT7   294   -----MQRTPPLLENSLPQCYQRVQQ--LQGVYSLQEQHFWTL-CSDVYVGTLKLIVAPD
EcYiiP  207   ---D--RALPD--E-ERQEIIDIVTS--WPGVSGAHDLRTRQSGPTRFIQIHLEM-----
SoYiiP  206   ALLD--RELDE--D-TRQRIKLIAKE--DPRVLGLHDLRTRQAGKTVFIQFHLEL-----
TtCrzB  198   GLMD--EGLPP--E-EVERIRAFLQERIRGRALEVHDLKTRRAGPRSFLEFHLVV-----
TmCzcD  206   --MDGMKRTEL--D-MYDDIFAVLER--FPNVHNPHRVRIRRVGTKYFIEMDIEV-----
CmCzcD  213   ----------D--DVDLAEVEKQILA--TPGVKSFHDLHIWAL-TSGKASLTVHV-----
PaCzcD  210   -------GVPK--EIQLAELREALLG--IPGVTGLHDLHVWSI-TSGKISLTSHL-----

                        ▼                      ▼ ▼   ▼
hZnT7   326   ADARWILSQTHNIFTQAGVRQ---------LYVQIDFAAM-------------   376
EcYiiP  252   -EDSLPLVQAHMVADQVEQAILRRFPGSD-VIIHQDPCSVV------------   290
SoYiiP  254   -DGNLSLNEAHSITDTTGLRVKAAFEDAE-VIIHQDPVQVEPTTQ--------   291
TtCrzB  248   -RGDTPVEEAHRLCDELERALAQAFPGLQ-ATIHVEPEGERKRTNP-------   291
TmCzcD  254   -DGKMSVKDAHELTVKIRKEMLKRRDDIEDVTIHVEPLGNVEEEGFGLKKGEKK   306
CmCzcD  253   -VNDTAVNPEMEVLPELKQMLADKFDITH-VTIQFEL----------------   285
PaCzcD  253   -VYDPALVDAEALLGTVKALLHDRYEIEH-STLQLETSACA------------   291
```

Supplementary Fig. 18 | Comparison of Zn^{2+}-binding residues between hZnT7 and other zinc transporters belonging to the CDF superfamily. Amino acid sequence alignment of the C-terminal regions of hZnT7 and bacterial Zn^{2+} transporters. The sequences were downloaded from the UniProt site for human ZnT7 (hZnT7, UniProt ID: Q8NEW0), *Escherichia coli* YiiP (EcYiiP, UniProt ID: P69380), *Shewanella oneidensis* YiiP (SoYiiP, UniProt ID: Q8E919), *Thermus thermophilus* CzrB (TtCzrB, UniProt ID: Q8VLX7), *Thermotoga maritima* CzcD (TmCzcD, UniProt ID: Q9WZX9), *Cupriavidus metallidurans* CzcD (CmCzcD, UniProt ID: P13512) and *Pseudomonas aeruginosa* CzcD (PaCzcD, UniProt ID: Q9I6A3). Residues involved in Zn^{2+} binding are indicated by black arrowheads and marked by green boxes. The corresponding residues in hZnT7 are colored pink.

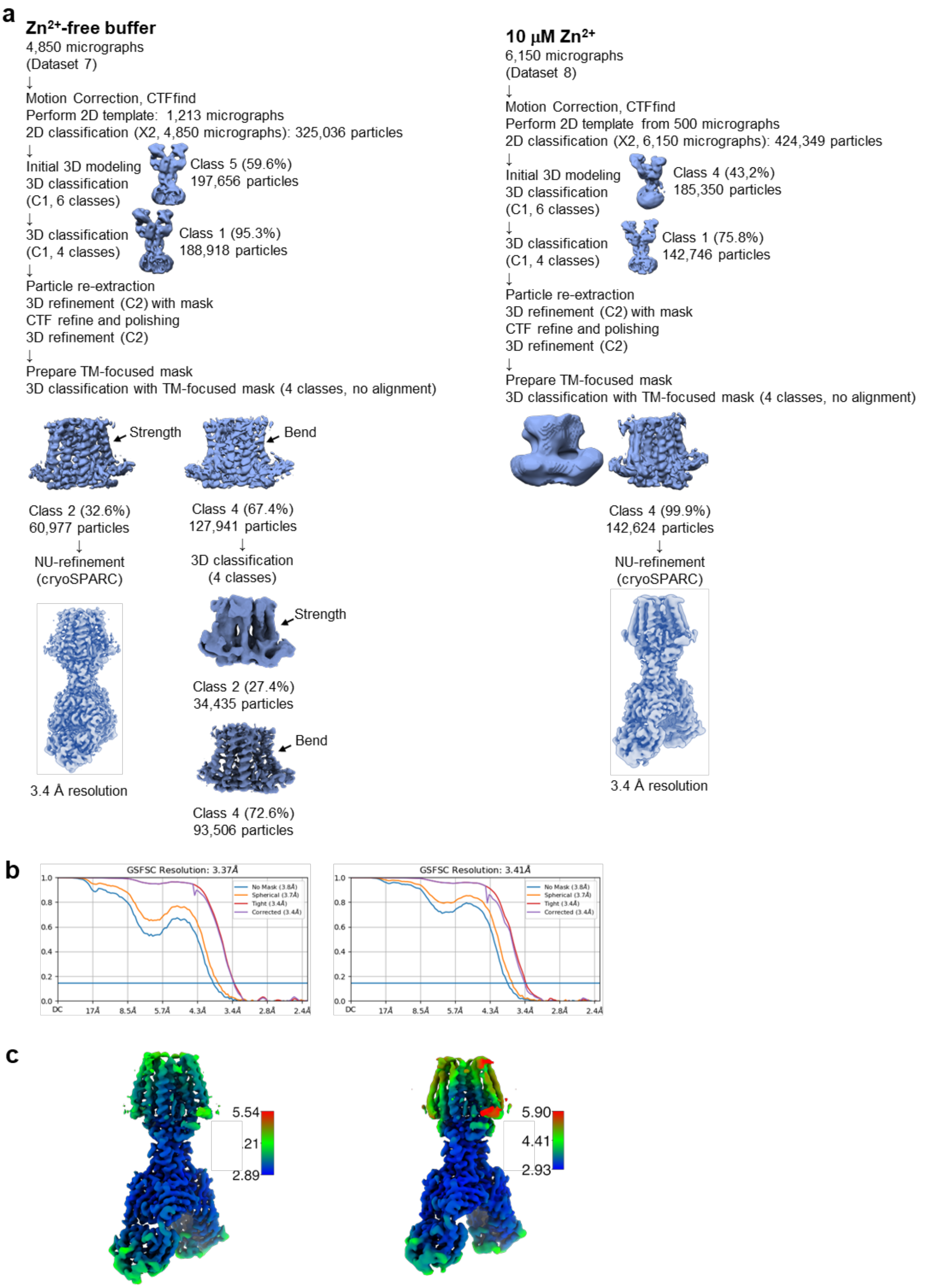

Supplementary Fig. 19 | Cryo-EM analysis of Zn^{2+}-unbound and Zn^{2+}-bound hZnT7ΔHis-loop in complex with Fab#1. a, Workflow of cryo-EM data processing and map refinement of the hZnT7ΔHis-loop (Zn^{2+}-unbound)-Fab#1 complex and the hZnT7ΔHis-loop (Zn^{2+}-bound)-Fab#1 complex. **b**, Gold-standard FSC plots of the hZnT7ΔHis-loop (Zn^{2+}-unbound)-Fab#1 complex (left) and the hZnT7ΔHis-loop (Zn^{2+}-bound)-Fab#1 complex (right) from cryoSPARC.

c, Local-resolution map of the hZnT7ΔHis-loop (Zn^{2+}-unbound)-Fab#1 complex (left) and the hZnT7ΔHis-loop (Zn^{2+}-bound)-Fab#1 complex (right) calculated by cryoSPARC. Bars on the right side indicate local resolution in Å.

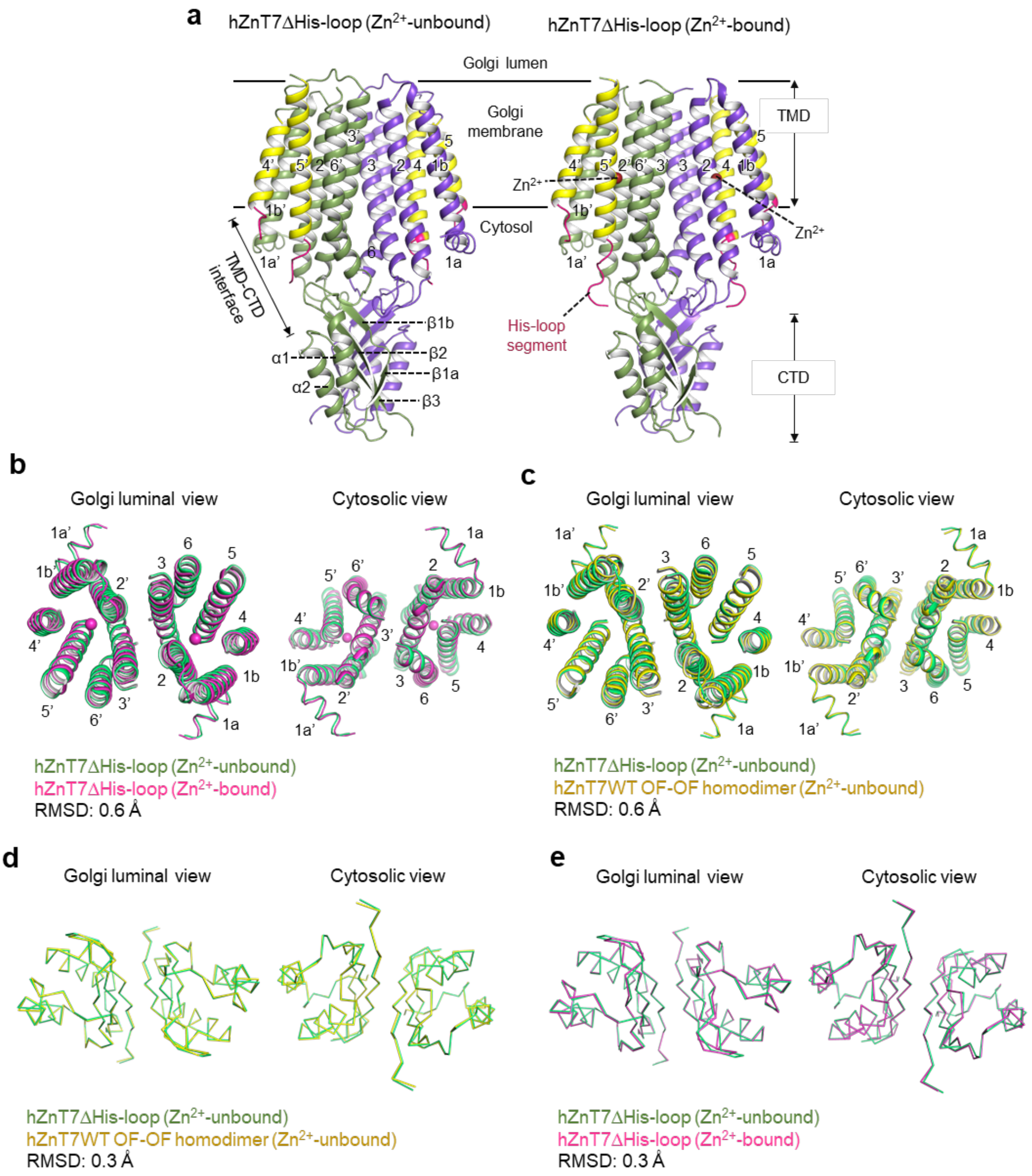

Supplementary Fig. 20 | Cryo-EM structures of hZnT7ΔHis-loop in Zn^{2+}-unbound and -bound states. a, Cartoon representation of the hZnT7ΔHis-loop in the Zn^{2+}-unbound (left) and -bound (right) states. Gray lines indicate the boundaries of the membrane. TM1 to TM3 and TM6 are shown in green or violet while TM4 and TM5 are shown in yellow. The visible His-loop segments are shown in hot-pink color. Numbers indicate the TM helix number from the N-terminus. Red spheres indicate Zn^{2+}. TMD, transmembrane domain; CTD, cytosolic domain. **b,** Superimposition of the TMD of hZnT7ΔHis-loop in the Zn^{2+}-unbound and -bound states. The CTD, Golgi luminal and cytosolic loops are not shown for clarity. **c,** Superimposition of the TM domain of hZnT7ΔHis-loop and hZnT7 WT in the Zn^{2+}-unbound state. The CTD, Golgi luminal and cytosolic loops are not shown for clarity. **d,** Superimposition of the CTD of the hZnT7ΔHis-

loop in the Zn^{2+}-unbound and -bound states. Only the CTD is shown for clarity. **e,** Superimposition of the CTD of hZnT7ΔHis-loop and hZnT7 WT in the Zn^{2+}-unbound state. Only the CTD is shown for clarity.

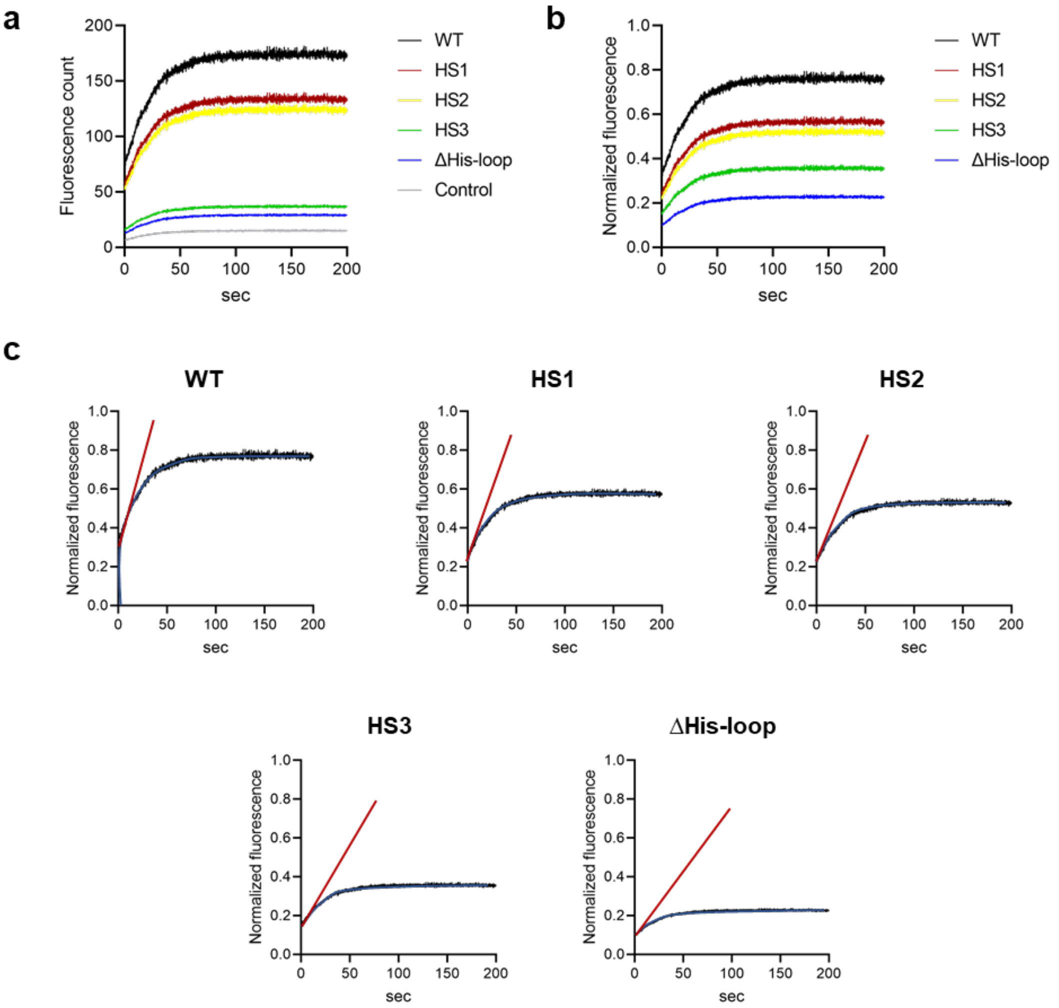

Supplementary Fig. 21 | Examples of the observed data for Zn^{2+} uptake by hZnT7 WT and its mutants. a, Representative fluorescence increase curves of the empty liposome (Control) and hZnT7 proteoliposomes (WT, HS1, HS2, HS3 and ΔHis-loop) after addition of Zn^{2+}. **b,** Representative normalized fluorescence increase curves of the hZnT7 proteoliposomes after addition of Zn^{2+}. For normalization, the maximal fluorescence intensity was measured after solubilizing the Fluozin-3-containing liposome or proteoliposomes with 1% octyl-β-D-glucoside detergent solution containing 0.4 mM $ZnSO_4$. Zn^{2+} transport activity was then calculated using the equation $(FP/FP_{max})–(FL/FL_{max})$ over time. FP, fluorescence counts of proteoliposomes; FL, fluorescence counts of liposome; FP_{max}; maximal fluorescence counts of proteoliposomes; FL_{max}, maximal fluorescence counts of liposomes. **c,** After subtracting the fluorescence signals of the empty liposome from those of proteoliposomes, the normalized fluorescence curves are fitted by a single exponential function (blue line), thus providing the initial rate of Zn^{2+} uptake at time 0 (red line).

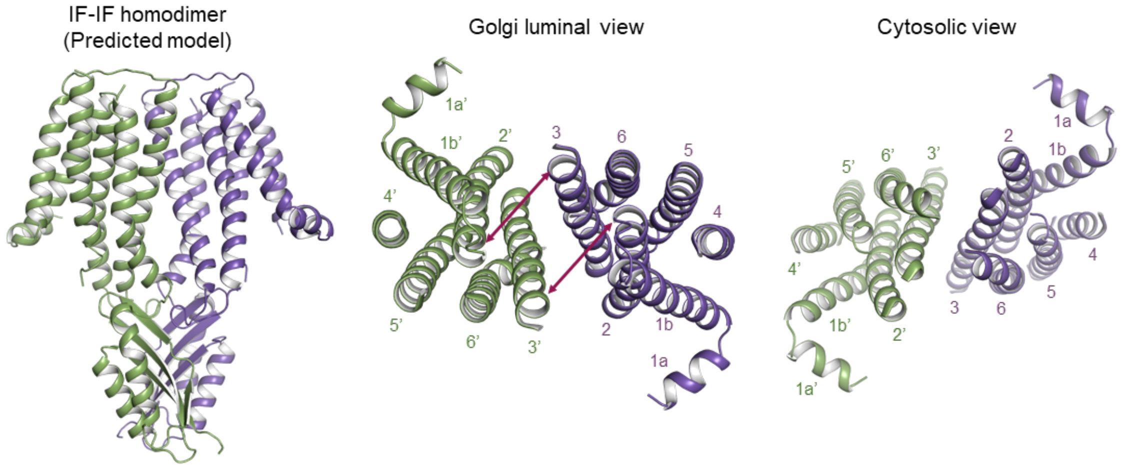

Supplementary Fig. 22 | hZnT7 IF-IF homodimer model predicted by GalaxyHomomer web server. Cartoon representation of a predicted model of the IF-IF homodimer of hZnT7 viewed from the side (left), Golgi lumen (middle) and cytosol (right). Numbers indicate the TM helix number from the N-terminus. Only the TMD is shown in the luminal and cytosolic views for clarity. The red double-headed arrows indicate the distance between the luminal ends of TM2 and TM3' (or TM2' and TM3) at the dimer interface. The IF protomer from the Zn^{2+}-unbound IF-OF heterodimer (IF-OF form) was used for modelling the IF-IF homodimer of hZnT7 in the GalaxyHomomer web server.

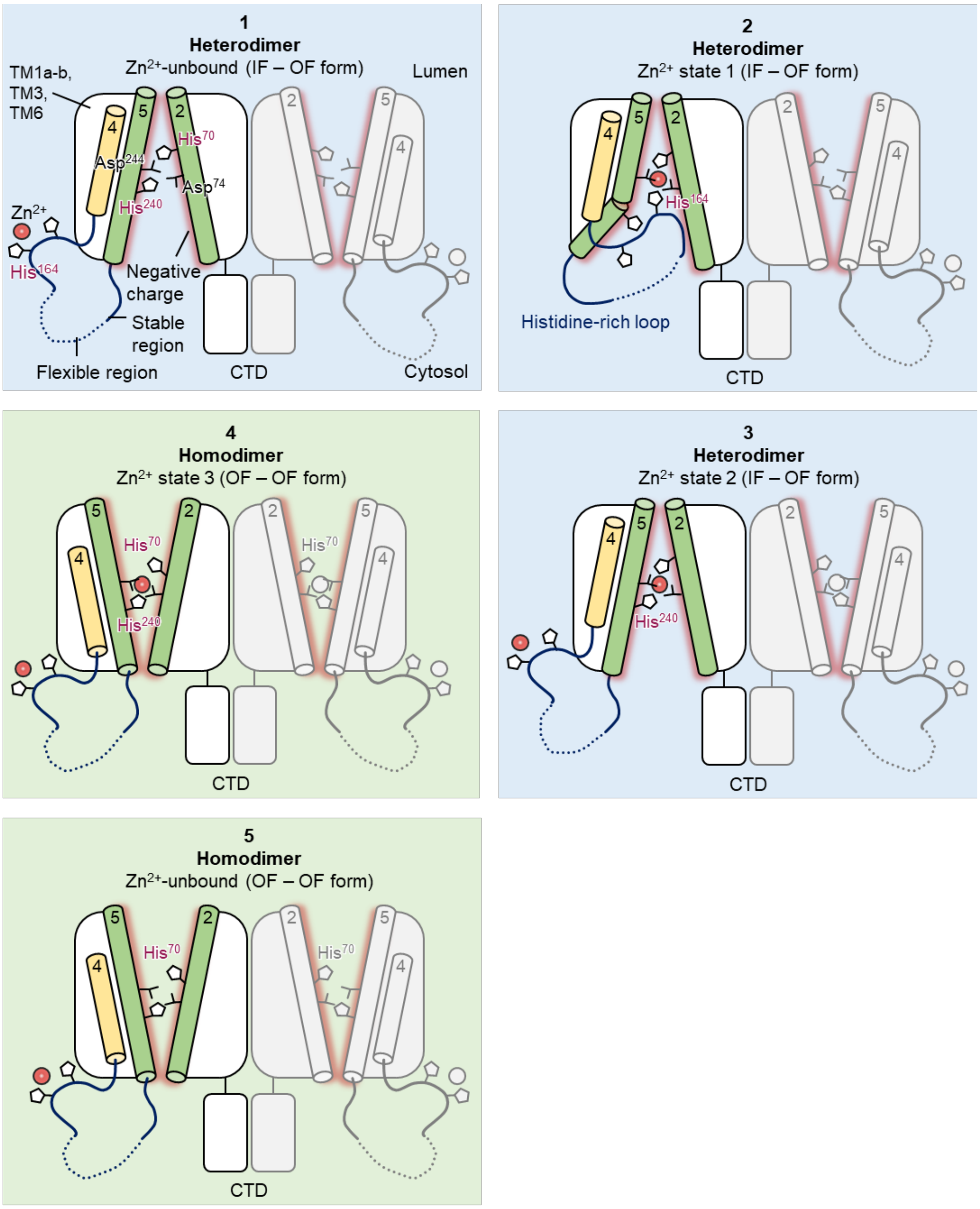

Supplementary Fig. 23 | A possible model of Zn^{2+} transport by the hZnT7 dimer, based on the cryo-EM structures of the hZnT7 dimers revealed in this work. Given the structures of another protomer (right one, shown in gray) revealed by the present cryo-EM analysis, the model has been modified from Fig. 7. However, a comprehensive view of the Zn^{2+} transport mediated by the hZnT7 dimer will require further structural information on other intermediates along the sequential steps of Zn^{2+} uptake from the cytosol and Zn^{2+} release to the Golgi lumen.

Supplementary Table 1 | Cryo-EM data collection, refinement, and validation statistics of hZnT7 WT.

	OF-OF form (Zn^{2+} unbound)	OF-IF form (Zn^{2+} unbound)	IF/Zn-OF (Zn^{2+} state 1)	IF/Zn-OF/Zn (Zn^{2+} state 2)	OF/Zn-OF/Zn (Zn^{2+} state 3)
**Data collection and processing**					
Magnification	60,000	60,000	60,000	60,000	60,000
Voltage (kV)	300	300	300	300	300
Electron exposure ($e^-/Å^2$)	50	50	50	50	50
Defocus range (μm)	-0.8 to -1.7	-0.8 to -1.7	-0.8 to -1.7	-0.8 to -1.7	-0.8 to -1.7
Pixel size (Å)	0.788	0.788	0.788	0.788	0.788
PDB code	8J7T	8J7V	8J80	8J7W	8J7U
EMDB	EMD-36048	EMD-36050	EMD-36055	EMD-36051	EMD-36049
Symmetry imposed	C2	C1	C1	C1	C1
Initial particle images (no.)	1,1909,756	1,1909,756	2,232,644	2,110,423	2,110,423
Final particle images (no.)	115,162	28355	105,351	113,211	120,625
Map resolution (Å)	2.21	2.79	2.68	2.92	3.12
FSC threshold	0.143	0.143	0.143	0.143	0.143
Map resolution range (Å)	1.90 – 5.40	2.40 – 6.00	2.30 – 5.30	2.30 – 5.30	2.50 – 7.00
**Refinement**					
Initial model used (PDB code)	-	-	-	-	-
Model resolution (Å)	2.4	2.7	2.6	2.8	3.0
FSC threshold	0.5	0.5	0.5	0.5	0.5
Model resolution range (Å)	-	-	-	-	-
Map sharpening *B* factor ($Å^2$)	-45	-40	-59	-60	-55
Model composition					
Non-hydrogen atoms	11140	11083	11172	11026	11005
Protein residues	1438	1432	1444	1425	1423
Ligands	0	0	1	2	2
B factors ($Å^2$)					
Protein	89.4	114.7	128.3	129.6	145.7
Ligand	-		167.2	255.6	214.5
R.m.s. deviations					
Bond lengths (Å)	0.004	0.004	0.005	0.004	0.004
Bond angles (°)	0.652	0.725	0.728	0.737	0.833
Validation					
MolProbity score	1.78	1.74	1.97	1.97	1.92
Clash score	8.94	7.08	6.76	7.85	8.64
Poor rotamers (%)	1.85	1.54	3.05	2.60	1.92
Ramachandran plot					
Favored (%)	97.5	96.7	96.5	96.5	96.3
Allowed (%)	2.5	3.3	3.5	3.5	3.7
Disallowed (%)	0.0	0.0	0.0	0.0	0

Supplementary Table 2 | Cryo-EM data collection, refinement, and validation statistics of hZnT7ΔHis-loop.

	Apo-hZnT7ΔHis-loop (OF-OF form)	Zn^{2+}-hZnT7ΔHis-loop (OF/Zn-OF/Zn form)
**Data collection and processing**		
Magnification	60,000	60,000
Voltage (kV)	300	300
Electron exposure (e^-/Å^2)	60	60
Defocus range (μm)	-0.6 to -1.6	-0.6 to -1.6
Pixel size (Å)	0.788	0.788
PDB code	8J7X	8J7Y
EMDB	EMD-36052	EMD-36053
Symmetry imposed	C2	C2
Initial particle images (no.)	565,468	678,799
Final particle images (no.)	60,977	142,624
Map resolution (Å)	3.37	3.40
FSC threshold	0.143	0.143
Map resolution range (Å)	2.89 – 5.50	2.93 – 5.90
**Refinement**		
Initial model used (PDB code)	-	-
Model resolution (Å)	3.4	3.4
FSC threshold	0.5	0.5
Model resolution range (Å)	-	-
Map sharpening *B* factor (Å^2)	-95.0	-95.0
Model composition		
Non-hydrogen atoms	11,136	11,008
Protein residues	1,424	1,426
Ligands	0	2
B factors (Å^2)		
Protein	40.5	87.8
Ligand	-	180.3
R.m.s. deviations		
Bond lengths (Å)	0.002	0.002
Bond angles (°)	0.483	0.523
Validation		
MolProbity score	1.36	1.58
Clash score	4.79	7.04
Poor rotamers (%)	0.16	0.16
Ramachandran plot		
Favored (%)	97.46	96.86
Allowed (%)	2.40	3.14
Disallowed (%)	0.14	0
