# Supplementary material for: Cryo-EM structures of human zinc transporter ZnT7 reveal the mechanism of Zn2+ uptake into the Golgi apparatus
Source: Nat Commun. 2023 Aug 8;14:4770. doi: 10.1038/s41467-023-40521-5 (PMC10409766; doi:10.1038/s41467-023-40521-5)
Supplement: Supplementary file 3 — Description of Additional Supplementary Files [file 41467_2023_40521_MOESM3_ESM.pdf]

# Description of Additional Supplementary Files

**File name: Supplementary Data 1**
**Description:** Primers used in this work.

**File name: Supplementary Data 2**
**Description:** Amino acid sequences of the heavy and light chains of Fab#1 used in this work.

**File name: Supplemental Movie 1**
**Description:** Conformational transition of the $Zn^{2+}$-unbound hZnT7 protomer during the OF-to-IF form conversion viewed from the Golgi lumen (first half) and cytosol (second half). Note that only the left protomer undergoes the OF-to-IF conversion.

**File name: Supplemental Movie 2**
**Description:** Conformational transition of the $Zn^{2+}$-bound hZnT7 protomer during the conversion from the OF to IF ($Zn^{2+}$ state 1) form. $Zn^{2+}$ is omitted for simplification. Note that only the left protomer undergoes the OF-to-IF conversion.

**File name: Supplemental Movie 3**
**Description:** Conformational transition of the hZnT7 protomer during the $Zn^{2+}$ transport from the cytosol to the Golgi lumen. For clarity, only TM2, TM4, and TM5 are highlighted in this video. Since a single His residue is unlikely to capture $Zn^{2+}$ efficiently, other His or Asp/Glu residues in the His-loop likely cooperate with His164 for $Zn^{2+}$ recruitment from the cytosol. In the OF conformation, His70 moves away from the $Zn^{2+}$-binding site possibly via its protonation to facilitate $Zn^{2+}$ release to the Golgi lumen.
