# Supplementary material for: Cryo-EM structures of human zinc transporter ZnT7 reveal the mechanism of Zn2+ uptake into the Golgi apparatus
Source: Nat Commun. 2023 Aug 8;14:4770. doi: 10.1038/s41467-023-40521-5 (PMC10409766; doi:10.1038/s41467-023-40521-5)
Supplement: Supplementary file 9 — Reporting Summary [file 41467_2023_40521_MOESM9_ESM.pdf]

nature portfolio

Corresponding author(s): Kenji Inaba

Last updated by author(s): Jul 19, 2023

Reporting Summary

Nature Portfolio wishes to improve the reproducibility of the work that we publish. This form provides structure for consistency and transparency in reporting. For further information on Nature Portfolio policies, see our Editorial Policies and the Editorial Policy Checklist.

Statistics

For all statistical analyses, confirm that the following items are present in the figure legend, table legend, main text, or Methods section.

n/a	Confirmed	
☐	☒	The exact sample size (*n*) for each experimental group/condition, given as a discrete number and unit of measurement
☐	☒	A statement on whether measurements were taken from distinct samples or whether the same sample was measured repeatedly
☐	☒	The statistical test(s) used AND whether they are one- or two-sided<br>*Only common tests should be described solely by name; describe more complex techniques in the Methods section.*
☒	☐	A description of all covariates tested
☒	☐	A description of any assumptions or corrections, such as tests of normality and adjustment for multiple comparisons
☐	☒	A full description of the statistical parameters including central tendency (e.g. means) or other basic estimates (e.g. regression coefficient) AND variation (e.g. standard deviation) or associated estimates of uncertainty (e.g. confidence intervals)
☐	☒	For null hypothesis testing, the test statistic (e.g. *F*, *t*, *r*) with confidence intervals, effect sizes, degrees of freedom and *P* value noted<br>*Give P values as exact values whenever suitable.*
☒	☐	For Bayesian analysis, information on the choice of priors and Markov chain Monte Carlo settings
☒	☐	For hierarchical and complex designs, identification of the appropriate level for tests and full reporting of outcomes
☒	☐	Estimates of effect sizes (e.g. Cohen's *d*, Pearson's *r*), indicating how they were calculated

Our web collection on statistics for biologists contains articles on many of the points above.

Software and code

Policy information about availability of computer code

Data collection	Electron Microscopy data was collected using SerialEM v3.7.
Data analysis	RELION v3.1, RELION v4.0, cryoSPARC v3.3, CTFFIND4 v4.1.5, UCSF pyem v0.5, CCP-EM v1.6.0, COOT v0.9.8.7, PHENIX v1.20.1, MolProbity (part of PHENIX v1.20.1), PyMOL v2.5.2, UCSF Chimera v1.17, UCSF ChimeraX v1.5, MAFFT v7 wed-sever, APBS-PDB2PQR web-sever, Origin 7 SR4 v7.0552 and GraphPad Prism v9.3.1

For manuscripts utilizing custom algorithms or software that are central to the research but not yet described in published literature, software must be made available to editors and reviewers. We strongly encourage code deposition in a community repository (e.g. GitHub). See the Nature Portfolio guidelines for submitting code & software for further information.

Data

Policy information about availability of data

All manuscripts must include a data availability statement. This statement should provide the following information, where applicable:

- Accession codes, unique identifiers, or web links for publicly available datasets
- A description of any restrictions on data availability
- For clinical datasets or third party data, please ensure that the statement adheres to our policy

The atomic coordinates of human ZnT7 have been deposited in the Protein Data Bank under accession codes 8J7T (Zn2+-unbound OF-OF form), 8J7U (Zn2+-bound OF/Zn-OF/Zn form, i.e. Zn2+ state 3), 8J7V (Zn2+-unbound IF-OF form), 8J80 (IF/Zn-OF form, i.e. Zn2+ state 1), 8J7W (IF/Zn-OF/Zn form, i.e. Zn2+ state 2), 8J7X (Zn2

+-unbound hZnT7ΔHis-loop in OF-OF form, i.e. Apo-hZnT7ΔHis-loop) and 8J7Y (Zn2+-bound hZnT7ΔHis-loop in OF/Zn-OF/Zn form, i.e. Zn2+-hZnT7ΔHis-loop). Cryo-EM density maps of hZnT7 have been deposited in the Electron Microscopy Data Bank under accession codes EMD-36048 (OF-OF form), EMD-36049 (OF/Zn-OF/Zn form, i.e. Zn2+ state 3), EMD-36050 (IF-OF form), EMD-36055 (IF/Zn-OF form, i.e. Zn2+ state 1), EMD-36051 (IF/Zn-OF/Zn form, i.e. Zn2+ state 2), EMD-36052 (Apo-hZnT7ΔHis-loop in OF-OF form) and EMD-36053 (Zn2+-hZnT7ΔHis-loop in OF/Zn-OF/Zn form). The source data underlying Figures 7d and Supplementary Figure 1c are provided as a Source Data file.

Research involving human participants, their data, or biological material

Policy information about studies with human participants or human data. See also policy information about sex, gender (identity/presentation), and sexual orientation and race, ethnicity and racism.

Reporting on sex and gender	Not applicable
Reporting on race, ethnicity, or other socially relevant groupings	Not applicable
Population characteristics	Not applicable
Recruitment	Not applicable
Ethics oversight	Not applicable

Note that full information on the approval of the study protocol must also be provided in the manuscript.

Field-specific reporting

Please select the one below that is the best fit for your research. If you are not sure, read the appropriate sections before making your selection.

☒ Life sciences ☐ Behavioural & social sciences ☐ Ecological, evolutionary & environmental sciences

For a reference copy of the document with all sections, see nature.com/documents/nr-reporting-summary-flat.pdf

Life sciences study design

All studies must disclose on these points even when the disclosure is negative.

Sample size	No sample size calculation was performed. The cryo-EM sample size was determined by the number and quality of collected micrographs and particles. The same size of each dataset is described in Supplementary Tables 1 and 2 and the EM data processing part of the Methods section. Concretely, ~4,500 to ~6,800 movies were collected; ~1.2 to ~2.3 million particles were used for 2D class averaging; and ~28,000 to ~110,000 particles were used for final 3D reconstruction refinements. For the ITC measurements, at least twice replicates were performed for each experiment. For the Zn2+ transport assay, at least three independent experiments were repeated.
Data exclusions	No data has been excluded.
Replication	Cryo-EM data were collected from multiple cryo-EM grids over several data collection sessions to achieve the high-resolution structure analysis, as described in this paper. For Zn2+ transport assay, we made at least three independent replicates for each hZnT7 construct. For ITC measurement, we made at least two independent replicates for each hZnT7 construct. For SEC shown in Supplementary Fig. 1a and c, the samples were reproducibly purified at least three times. All attempts at replication were successful.
Randomization	This study did not include experiments with experimental group allocation, hence no randomization was attempted or needed in this study.
Blinding	This study did not include experiments with experimental group allocation, hence no blinding was attempted or needed in this study.

Reporting for specific materials, systems and methods

We require information from authors about some types of materials, experimental systems and methods used in many studies. Here, indicate whether each material, system or method listed is relevant to your study. If you are not sure if a list item applies to your research, read the appropriate section before selecting a response.

Materials & experimental systems

n/a	Involved in the study
☐	☒ Antibodies
☐	☒ Eukaryotic cell lines
☒	☐ Palaeontology and archaeology
☐	☒ Animals and other organisms
☒	☐ Clinical data
☒	☐ Dual use research of concern
☒	☐ Plants

Methods

n/a	Involved in the study
☒	☐ ChIP-seq
☒	☐ Flow cytometry
☒	☐ MRI-based neuroimaging

Antibodies

Antibodies used	To determine the cryo-EM structures of human ZnT7 in complex with Fab, we used our house-made monoclonal antibody that specifically binds the hZnT7 sample. In ELISA experiments, the serum was diluted in the range of 3/10,000,000 to 1/1.000.
Validation	The ability of our house-made monoclonal antibody to bind human ZnT7 was assessed by ELISA and size-exclusion chromatography.

Eukaryotic cell lines

Policy information about cell lines and Sex and Gender in Research

Cell line source(s)	HEK293T (ATCC, Cat#CRL-3216)
Authentication	None of the cell lines used have been authenticated.
Mycoplasma contamination	The cell lines used have been tested for mycoplasma contamination by the providers (negative results) but have not been retested in the lab.
Commonly misidentified lines (See ICLAC register)	No commonly misidentified lines were used in this study.

Animals and other research organisms

Policy information about studies involving animals; ARRIVE guidelines recommended for reporting animal research, and Sex and Gender in Research

Laboratory animals	6-weeks old female MRL/lpr mice maintained at temperature and humidity ranges of 22 to 26-degree celsius and 40% to 60%, respectively, under a 12-h light and 12-h dark cycle were used for preparation of the monoclonal antibody against hZnT7.
Wild animals	No wild animals were used in this study.
Reporting on sex	Female MRL/lpr mice were used for preparation of the monoclonal antibody against hZnT7. Sex-based analysis was not performed in this study.
Field-collected samples	No field collected samples were used in this study.
Ethics oversight	All animal experiments conformed to the guideline of the Guide for the Care and Use of Laboratory Animals of Japan, and were approved by the Kyoto University Animal Experimentation Committee.

Note that full information on the approval of the study protocol must also be provided in the manuscript.
